# ATG5 Is Essential for ATG8-Dependent Autophagy and Mitochondrial Homeostasis in *Leishmania major*

**DOI:** 10.1371/journal.ppat.1002695

**Published:** 2012-05-17

**Authors:** Roderick A. M. Williams, Terry K. Smith, Benjamin Cull, Jeremy C. Mottram, Graham H. Coombs

**Affiliations:** 1 Strathclyde Institute of Pharmacy and Biomedical Sciences, University of Strathclyde, Glasgow, United Kingdom; 2 Schools of Biology & Chemistry, The University of St. Andrews, St. Andrews, United Kingdom; 3 Wellcome Trust Centre for Molecular Parasitology, Institute of Infection, Immunity and Inflammation, College of Medical, Veterinary and Life Sciences, University of Glasgow, Glasgow, United Kingdom; University of California, Los Angeles, United States of America

## Abstract

Macroautophagy has been shown to be important for the cellular remodelling required for *Leishmania* differentiation. We now demonstrate that *L. major* contains a functional ATG12-ATG5 conjugation system, which is required for ATG8-dependent autophagosome formation. Nascent autophagosomes were found commonly associated with the mitochondrion. *L. major* mutants lacking ATG5 (Δ*atg5*) were viable as promastigotes but were unable to form autophagosomes, had morphological abnormalities including a much reduced flagellum, were less able to differentiate and had greatly reduced virulence to macrophages and mice. Analyses of the lipid metabolome of Δ*atg5* revealed marked elevation of phosphatidylethanolamines (PE) in comparison to wild type parasites. The Δ*atg5* mutants also had increased mitochondrial mass but reduced mitochondrial membrane potential and higher levels of reactive oxygen species. These findings indicate that the lack of ATG5 and autophagy leads to perturbation of the phospholipid balance in the mitochondrion, possibly through ablation of membrane use and conjugation of mitochondrial PE to ATG8 for autophagosome biogenesis, resulting in a dysfunctional mitochondrion with impaired oxidative ability and energy generation. The overall result of this is reduced virulence.

## Author Summary

Leishmaniasis is a disease of humans that is of major significance throughout many parts of the world. It is caused by the protozoan parasite *Leishmania* and mammals are infected through the bite of a sand fly in which the parasite develops. Parasite remodelling crucial for generation of the human-infective forms is aided by the catabolic process known as autophagy in which cell material is packaged within organelles called autophagosomes and subsequently broken down in the digestive lysosomal compartment. Here we show that autophagy in *Leishmania* requires the coordinated actions of two pathways, one of which involves a protein called ATG5. We have generated parasite mutants lacking this protein and shown that ATG5 is required for both autophagosome formation and also maintenance of a fully functional mitochondrion. The mutants lacking ATG5 have increased mitochondrial mass and phospholipid content, high levels of oxidants and reduced membrane potential, all being hallmarks of a dysfunctional mitochondrion with impaired ability for energy generation. Our results have thus revealed that a functional autophagic pathway is crucial for phospholipid homeostasis and mitochondrial function in the parasite and important for the parasite's differentiation, infectivity and virulence to its mammalian host.

## Introduction

*Leishmania* are widespread and important parasites of humans and dogs that produce a spectrum of diseases collectively called the leishmaniases. Differentiation between the three distinctive morphological forms, the procyclic promastigote, metacyclic promastigote and amastigote, is crucial for progression through the parasite's digenetic life cycle and requires extensive remodelling of its cellular constituents, a process in which the macroautophagic pathway is involved [1], [2].

**Figure 1:**
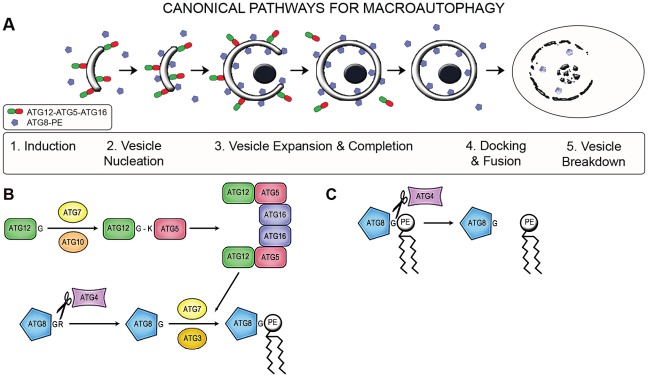
Canonical pathways of macroautophagy. (A) Autophagosome biogenesis and fate. The successive events during the generation of autophagosmes are depicted, from induction to breakdown. The involvement of ATG5, ATG12 and ATG8 and the two conjugation pathways, and the sequence in which they act, are depicted. (B) The two conjugation pathways involved in autophagosome biogenesis. The ATG12-ATG5-ATG16 complex formed in the first pathway is involved in the attachment of ATG8 to phosphatidylethanolamine (PE) during the second pathway. These processes in *Leishmania* differ from those of mammals in that the *Leishmania* ATG12 has an extended C-terminal domain beyond the glycine residue required for conjugation to ATG5, suggesting cleavage is required. In addition, *Leishmania* possess two ATG4s, which may act at different stages of autophagosome formation. (C) Cleavage of ATG8 from the surface of mature autophagosomes before they fuse with the lysosomal network, showing the second step involving ATG4.

Macroautophagy (hereafter autophagy) is a catabolic system that degrades and recycles organelles and proteins [3]–[5]. In yeast and mammals, two ubiquitin-like conjugation systems, involving ATG8 and ATG12 respectively, are normally required for autophagosome formation although other mechanisms (non-canonical autophagy) have recently been recognised [6]. These two conjugation systems also utilise proteins encoded by six of the thirty-two known autophagy genes (designated *ATG*) with the conjugation of ATG8 to phosphatidylethanolamine (PE) occurring in one pathway and ATG12 to ATG5 in the other (see Figure 1).

In the ATG5-ATG12 pathway, ATG12 is activated by the E1-like enzyme ATG7, a thioester bond is formed between the carboxyl of its C-terminal glycine residue and the active cysteine of ATG7 [7]. ATG12 is then transferred to the active cysteine residue of the E2-like enzyme ATG10 [8] and subsequently to the ε-amino group of a conserved lysine residue of ATG5; an isopeptide bond with the exposed glycine residue of ATG12 being formed [5]. This process requires ATP and the complex subsequently interacts with the ATG6-Vps34 complex, ATG2, ATG14, ATG16, ATG18 and ATG21, proteins on the pre-autophagosomal membrane known as the phagophore [9]. The ATG5-ATG12 complex is crucial for the curvature of the phagophore in canonical autophagy. Work with mammalian cells and yeast has suggested that the phagophore is initially formed by membrane invagination of the centre of a phosphatidylinositol-3-phosphate (PI3P)-enriched spot, called the omegasome, formed by the action of phosphatidylinositol kinase, Vps34, on PI3P [10]. Cell membranes are now known to be involved in autophagosome initiation and the endoplasmic reticulum (ER), mitochondria, plasma membrane and Golgi apparatus have all been implicated [11]–[13].

It is to this developing phagophore that the attachment of ATG8-PE occurs; a key event for autophagosome formation. This is after the C-terminus of the precursor ATG8 has been cleaved by the ATG4 cysteine peptidase, to expose a C-terminal glycine. It is this glycine that is conjugated to PE through the catalytic actions of the E1-like and E2-like enzymes ATG7 and ATG3, respectively. The ATG5-ATG12 complex also contributes to this lipidation of ATG8 to PE through its E3-like activity which enhances the activity of ATG3 [14]; this reinforces the role of the complex in autophagosome biogenesis. The origin of the PE required for this process has been considered to be the ER in most mammalian cells [11], although it was recently shown to be the mitochondrion in mammalian cells under starvation conditions [12]. ATG8 incorporation onto the phagophore marks the start of cargo recruitment and acquisition. Adaptor proteins such as p62 and NIX attached to protein aggregates and damaged organelles, respectively, bind to ATG8-PE embedded on the nascent autophagosome [15], [16].

In time, the phagophore expands and there is closure of the autophagic membranes (with the cargo contained therein), processes that rely upon the ability of ATG8-PE to oligomerise and form aggregates and hemifusions [17]. The ATG5-ATG12 complex dissociates from the nascent autophagosome just before or after the nascent autophagosome buds off the omegasome with closure via a zippering mechanism [5]. For delivery and degradation of the autophagosome to the lysosome or vacuole and subsequent degradation of the contents, there is the requirement for ATG8 on the outer membrane of the autophagosome to be cleaved by ATG4 from its anchoring PE to facilitate fusion of the autophagosome with the endosomal and lysosome systems.

Some analyses of the genome of *L. major* suggested that the mechanism of autophagy in *Leishmania* may differ from that in other mammals and yeast in that genes encoding proteins required for the ATG5-ATG12 conjugation pathway appeared to be absent; this prompted speculations that this conjugation pathway may have evolved relatively recently [18]. These *in silico* findings also lead to the hypothesis that an alternative process known microautophagy may be especially important in these protozoa, which was supported in a report on glycosome turnover [19]. Nevertheless, in our previous studies we showed that autophagy involving ATG8 lipidation to PE occurs in *Leishmania* [1], [2] and that *L. major* does have genes that encode proteins with some apparent similarity to ATG5, ATG7, ATG10 and ATG12 [20]. Thus one objective of this study was to test experimentally the hypothesis that these proteins constitute a canonical ATG5-ATG12 conjugation pathway that is a key component of autophagy in *Leishmania* and to further characterise the pathway itself.

One of the functions of autophagy is recycling organelles including peroxisomes (pexophagy) and mitochondria (mitophagy). The mitochondrion is required for energy production *via β*-oxidation and oxidative phosphorylation but is also potentially able to regulate cell signalling pathways, maintain calcium and phospholipid levels, and promote cell death *via* apoptosis [21]. Thus its homeostasis is vital and autophagy is thought to have some role in this [22]. Evidence for interplay between autophagy and mitochondria has been increasing in recent years [23] with reports of mitochondrial function being compromised in the absence of a functional autophagic pathway [24], [25] and mitochondria regulating autophagy *via* signalling pathways [26]. However, the full mechanisms mediating this interplay are not understood fully. Mitophagy, which involves engulfment of the damaged mitochondrion into an autophagosome [22], [27], [28], has not been reported in *Leishmania* and the presence of a single mitochondrion, albeit comprising a large complex network, in the parasite raises questions on whether mitophagy *per se* can occur and if so how. Thus a second aim of this study was to elucidate the extent to which autophagy plays a role in mitochondrion homeostasis, with the hypothesis that the unitary mitochondrion may well necessitate interactions that differ from those that occur in mammalian cells and yeast.

PE is crucial for the binding of ATG8 in the formation of autophagosomes, but more generally it is a major component of biological membranes, especially mitochondrial membranes, and is involved in a wide range of biological processes from cell signalling, cell division, membrane fusion and trafficking events [29], [30]. There are two main routes known for PE synthesis, the Kennedy pathway and *via* phosphatidylserine decarboxylase (PSD). The latter occurs in the mitochondria in typical eukaryotes, utilising translocated phosphatidylserine (PS) synthesised in the ER [30]. However, it is thought to be insignificant in PE synthesis in *Leishmania* [31], [32], although present in the *Leishmania* mitochondrion [33], because sphingolipid metabolism in *Leishmania* is, unlike the situation in mammals, intrinsically linked with PE metabolism and provides the Kennedy pathway, which appears to terminate in the mitochondrion in trypanosomatids, with ethanolamine-phosphate [31], [32]. Thus the evidence as far as it stands for *Leishmania* suggests that PE is synthesised in the single mitochondrion before being distributed to other cell membranes. Therefore we hypothesised that the PE required for autophagosome formation may all be obtained from the mitochondrion directly in *Leishmania*, unlike the situation in most mammalian cells and yeast under normal conditions.

Thus this study was founded on the concept that the unusual nature of *Leishmania* in terms of mitochondrial structure and phospholipid biosynthesis distinguishes it from mammalian cells and makes it an interesting organism in which to study the interplay, if any, between autophagy and the mitochondrion. Our experimental approach to test the various hypotheses was to generate mutants lacking *ATG5*, and analyse the phenotype of the resulting mutant. This has not only allowed analysis of the interplay between autophagy and mitochondrial homeostasis but also the importance of autophagy for parasite viability, differentiation and virulence. The findings show clear correlation between autophagy and mitochondrial homeostasis and suggest that one contribution of autophagy to this is maintenance of appropriate PE composition in the mitochondrion. A consequence of the changes is markedly reduced virulence.

## Results

### Reconstitution of the ATG12–ATG5 conjugation pathway *in vitro*

**Figure 2:**
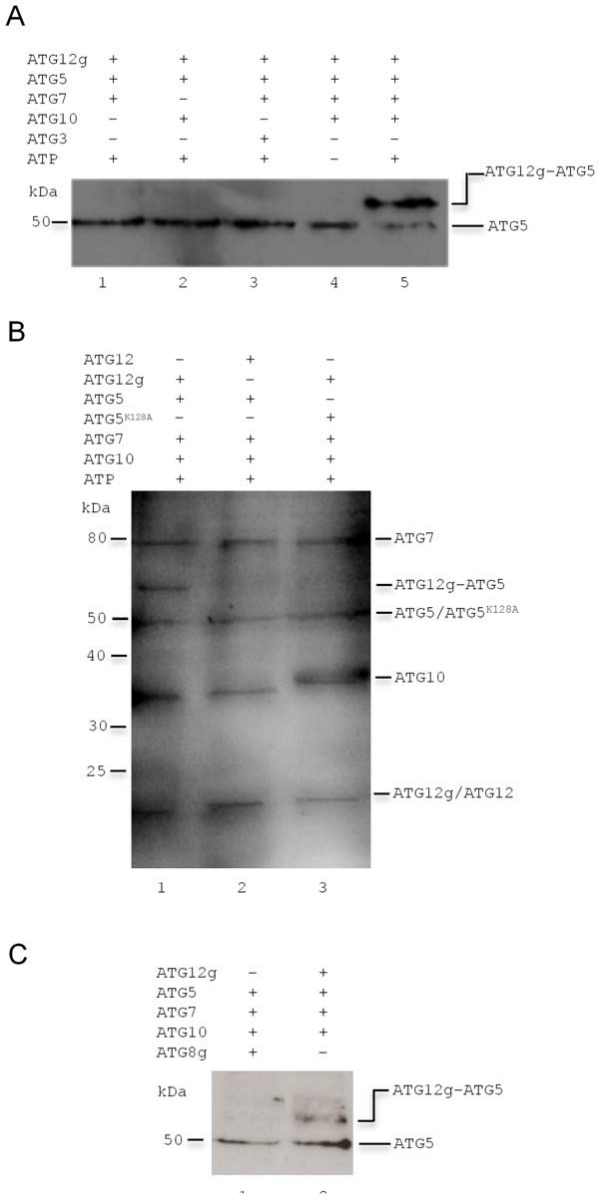
Reconstitution of the *L. major* ATG5-ATG12 conjugation system *in vitro*. (A) Recombinant proteins were mixed, as indicated, in the presence (lane 5) or absence (lane 4) of ATP and incubated for 1 h at 30°C before being separated by SDS-PAGE and analyzed by western blotting using the α-His antibody. Components present (+) or absent (−) from the assay are indicated. (B) Recombinant proteins were mixed, as indicated, and incubated as above. The reactions were stopped, subjected to SDS-PAGE and stained with Coomassie Blue. (C) Recombinant proteins were mixed, as indicated, incubated as above and analyzed with α-His antibody.

We have previously shown using western blot analysis of *Leishmania* lysates with an ATG12-specific antibody that ATG12 exists in two forms, one corresponding to the molecular mass of ATG12 and a second of higher molecular mass that was predicted to be an ATG5-ATG12 conjugate [20]. To provide further evidence that *Leishmania* has an ATG5-ATG12 conjugation system, we expressed and purified ATG5, ATG7, ATG10 and ATG12 recombinant proteins and analysed their ability to catalyse the formation of an ATG5-ATG12 conjugate in a reconstitution assay similar to those described previously [14], [34] The purified recombinant ATG7, ATG10 and a mutant ATG12 terminating at the scissile glycine (and named ATG12g; see ref [20]) were mixed with histidine-tagged ATG5 and ATP. Western blot analysis of the resultant mixture with α-His antibody detected the 50 kDa ATG5 and 70 kDa ATG12g-ATG5 conjugate (Figure 2A, lane 5). Analysis of the 70 kDa protein by mass spectrometry identified peptide fragments of both ATG5 and ATG12. The omission of ATG10 (Figure 2A, lane 1), ATG7 (Figure 2A, lane 2) or ATP (Figure 2A, lane 4) abolished the formation of the ATG5-ATG12 conjugate, suggesting that all the components were required. Further, no ATG5-ATG12 conjugate was formed when ATG10 was replaced with ATG3 (Figure 2A, lane 3), showing that in this assay there is no functional redundancy between the two *L. major* E2 enzymes ATG10 and ATG3. In total, the data suggest that recombinant ATG5, ATG7, ATG10 and ATG12g comprise the protein components required to form the ATG5-ATG12 conjugate in *L. major*, and the process is energy-dependent.

To determine if formation of the ATG5-ATG12 conjugate required lys^128^ of ATG5 and the terminal gly^185^ of ATG12, we prepared recombinant ATG12 and ATG12g and recombinant ATG5 in two forms - native and a mutant form with the lys^128^ substituted by ala (designated ATG5 and ATG5^K128A^, respectively). Western blot analysis confirmed that a constitution assay mix with *L. major*'s ATG12g and native ATG5 formed the ATG5-ATG12 conjugate (Figure 2B, lane 1), whilst the native ATG12 and native ATG5 (Figure 2B, lane 2) and the ATG12g and ATG5^K128A^ (Figure 2B, lane 3) did not. The lack of activity of ATG5^K128A^ in the assay is excellent evidence that it is specific for ATG5 itself and is not promiscuous. In addition, as the *L. major* ATG12 has a key ATG8-like feature (a C-terminal extension beyond the scissile glycine that requires processing before conjugation), we replaced the truncated ATG12g with a similarly truncated ATG8g in the reconstitution assay. However, no ATG5-ATG8 conjugate could be detected (Figure 2C, lane 1) whereas the control experiment with ATG12g under the same conditions formed the ATG5-ATG12 conjugate (Figure 2C, lane 2). These data confirm the functional difference between the proteins, which we had putatively identified as ATG8 and ATG12. Overall, these results suggest that the ATG5-ATG12 conjugate is formed by a reaction between the exposed glycine residue of ATG12 and the ε-amino group of lys^128^ of ATG5. The results also indicate ATG7 and ATG10 function as E1 and E2 enzymes, respectively. In addition, they show that the native ATG12 in *Leishmania* needs processing to enable it to function - a control mechanism that is not present in ATG12 from yeast or higher eukaryotes; the enzyme mediating this cleavage is unknown.

### Occurrence of ATG5-labelled puncta in *Leishmania* promastigotes

Previously we showed that ATG12 and ATG8 co-localized in *L. major*, but that most ATG8-containing autophagosomes lacked ATG12 [20]. This was consistent with *L. major* ATG12 being associated with nascent phagophores, but not fully formed autophagosomes containing cargo. To investigate the occurrence and location of the ATG5-ATG12 conjugate, we have now studied co-localisation of the two proteins in living promastigotes. mCherry-ATG5 (mC-ATG5) and green fluorescent protein-ATG12 (GFP-ATG12) were expressed singly and also co-expressed in *L. major* promastigotes and the resulting lines were analysed by fluorescence microscopy.

**Figure 3:**
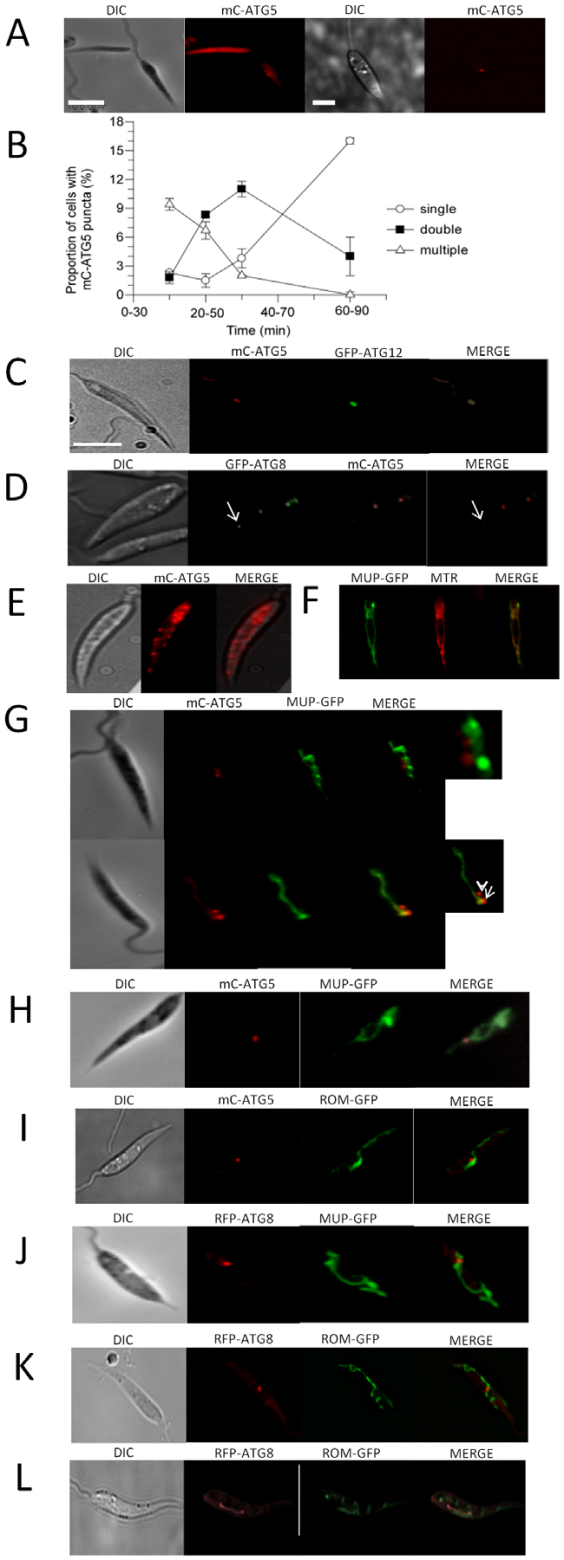
ATG5 puncta in *L. major* promastigotes. (A) The occurrence of mC-ATG5 puncta in WT *L. major* promastigotes expressing mC-ATG5 incubated in nutrient-rich medium at log phase at 26°C. (B) The multiplicity of puncta in promastigotes expressing mC-ATG5 after starvation for up to 1 h in PBS at 26°C. The time ranges indicated reflect the starvation period together with the 30 min period during which the observations on the microscope were undertaken. (C) Co-labelling of puncta with mC-ATG5 and GFP-ATG12 co-expressed in promastigotes incubated in PBS for 2 h at 26°C. (D) In nutrient-rich medium, GFP-ATG8 punctum without mC-ATG5 staining is arrowed. (E) Incubated in PBS for 30 min at 26°C. (F) In nutrient-rich medium at 26°C. (G) Incubated in PBS for 30 min at 26°C, the small panels are enlargements of the merged panels. (H–I) Promastigotes in nutrient-rich medium. (J–K) Promastigotes at late log phase in nutrient-rich conditions. (L) Promastigotes at stationary phase in nutrient-rich conditions. Scale bar throughout, 10 µm.

When grown in nutrient-rich medium and at early logarithmic growth phase, most cells expressing mC-ATG5 had the fluorescence evenly distributed throughout the cytoplasm (Figure 3A, left panels) with only 2% having a single mC-ATG5-labelled punctum in the cytosol (Figure 3A, right panels). However, under starvation conditions for an hour or more (known to induce autophagy [1]) 20±3% of the cells had mC-ATG5 puncta and of these 80±2% had just one (Figure 3B). All of the puncta in cells with both mC-ATG5 and GFP-ATG12 contained both labels (Figure 3C). To investigate co-localisation of ATG5 and ATG8, mC-ATG5 and GFP-ATG8 were co-expressed in promastigotes and late logarithmic stage cells analysed for puncta by fluorescence microscopy. Of the puncta in the promastigotes, 60±16% of mC-ATG5-labelled puncta also contained GFP-ATG8 but only 31±6% of GFP-ATG8-labelled puncta also had mC-ATG5 (Figure 3D).

The dynamics of the appearance of the mC-ATG5-labelled puncta was studied by using shorter starvation incubation periods. This showed that there was an early phase of up to 30 min starvation when several puncta were observed before the number declined (Figs. 3B and 3E). With this period of starvation, promastigotes expressing either GFP-ATG8 or GFP-ATG12 alone did not have puncta. These data are consistent with ATG5 being the first of these proteins to become recruited when the biogenesis of autophagosomes is initiated.

Interestingly, the early puncta were distributed around the promastigotes (Figure 3E) in a way consistent with the distribution of the typically reticulate mitochondrion that is present in the cells. This prompted us to look for co-localisation between nascent autophagosomes and the mitochondrion. We used two mitochondrial proteins as markers for the mitochondrion, the ubiquitin-like peptidase MUP (LmjF26.2070) and the serine peptidase rhomboid (LmjF04.0850). MUP fused to GFP was used as a marker for the outer membrane of the mitochondrion, as the mammalian homologue of MUP is located on the surface of mitochondria [35] and we showed that MUP-GFP co-localised with MitoTracker Red in *Leishmania* (Figure 3F). When MUP-GFP was co-expressed with mC-ATG5, more than half of the puncta after 30 min starvation were associated with the mitochondrion (Figure 3G). Differentiation of the procylic promastigote form to the metacyclic promastigote form in nutrient-rich conditions produced a localization profile for mC-ATG5 similar to that described for cells after 1 h starvation, with 19±3% of the cells having puncta of which 66±16% were in association with the mitochondrion (Figure 3H). The serine peptidase rhomboid is predicted to be located in the inner mitochondrial membrane [36], so we expressed rhomboid-GFP (ROM-GFP) and confirmed it as a second mitochondrial marker by co-localisation with MitoTracker Red (MTR, data not shown). Co-expression of this and mC-ATG5 revealed that 62±25% of the ATG5-labelled puncta were associated with the mitochondrion (Figure 3I).

We also looked for association of ATG8-labelled puncta with the mitochondrion. Fluorescence microscopy of late log phase promastigotes expressing both MUP-GFP and red fluorescent protein-ATG8 (RFP-ATG8) showed that 55±3% had ATG8-labelled puncta of which 60±9% were in apparent association with the mitochondrion (Figure 3J). Equivalent experiments with ROM-GFP rather than MUP-GFP gave similar data with, on average, 53±4% of the ATG8-labelled puncta being associated with the mitochondrion (Figure 3K). However, in no case was MUP-GFP or ROM-GFP fluorescence detectable within RFP-ATG8-labelled puncta, nor were the labelled MUP or ROM detectable with a RFP-ATG8-labelled elongated structure sometimes appearing in stationary phase promastigotes and thought likely to be the MVT-lysosome ([1] and Figure 3L). Relocation of mitochondrial components to the lysosome is one assay for mitophagy in yeast [37]. These data together suggest that the processes that we observed under the conditions of our experiments were not mitophagy.

Our observations are consistent with a large proportion of the phagophore biogenesis being initiated at the mitochondrial membrane with the involvement of ATG5 and ATG12, with subsequent recruitment of ATG8 as the nascent autophagosomes develop; these then lose ATG5 and the autophagosomes become located in the cytosol.

### *ATG5* is essential for autophagosome formation

**Figure 4:**
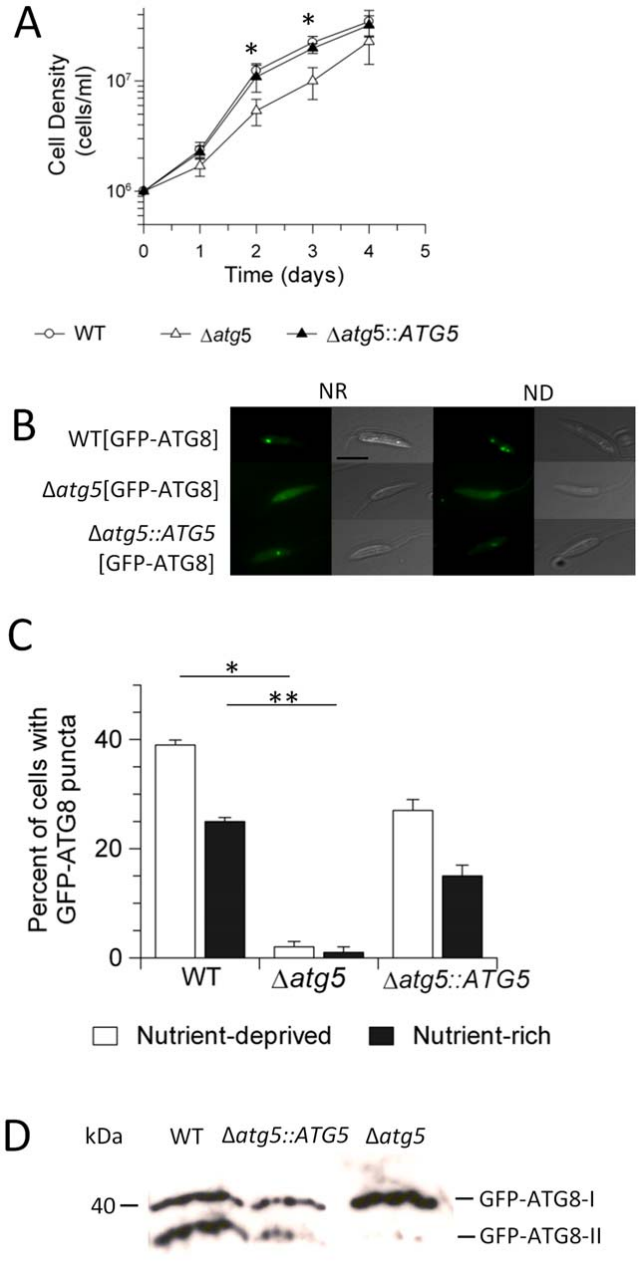
Phenotypic characterisation of Δ*atg5*. (A) Growth curve of *Leishmania* promastigotes in HOMEM medium at 26°C. *, Δ*atg5* differed significantly from WT (p<0.05). (B) The occurrence of GFP-ATG8 puncta in promastigotes after incubation in nutrient-deprived (PBS, ND) and nutrient-rich (HOMEM medium, NR) conditions for 2 h at 26°C. Scale bar, 10 µm. (C) Occurrence of GFP-ATG8 puncta in promastigotes when incubated in the conditions detailed in (B). Means ± SD from four independent experiments. * and **, occurrence of GFP-ATG8 puncta in Δ*atg5* were significantly different from in WT in nutrient-deprived and nutrient-rich conditions (p<0.05). (D) Western blot analysis of extracts of promastigotes expressing GFP-ATG8 at logarithmic growth under standard conditions and probed with α-GFP antibody. The faster migrating, lipidated band is labelled GFP-ATG8-II while the un-lipidated band migrating more slowly is labelled GFP-ATG8-I.

In order to investigate further the involvement of ATG5 in the parasite, promastigote mutants lacking both copies of the *ATG5* gene were generated by homologous recombination and verified by Southern blot analysis (Figure S1). An add-back line was generated by integrating *ATG5* with an N-terminal 6× histidine tag into the ribosomal locus. These cloned lines were named Δ*atg5* and Δ*atg5*::*ATG5*, respectively, and were used to infect mice from which the parasites were re-isolated to provide promastigotes for phenotypic analysis. The growth rate of Δ*atg5* promastigotes was reduced compared with the wild type (WT) and the add-back lines (Figure 4A). Δ*atg5* were unable to form GFP-ATG8 labelled autophagosomes in either nutrient-rich media or under starvation conditions (Figs. 4B–C), and there was very little conjugation of GFP-ATG8 to PE to generate GFP-ATG8-PE (designated GFP-ATG8-II, Figure 4D; see [1]); consistent with the cells being incapable of forming autophagosomes. Together, these findings show that ATG5 is crucial for autophagosome biogenesis and autophagy in *Leishmania*.

### *ATG5*-deficient promastigotes have a dysfunctional mitochondrion

**Figure 5:**
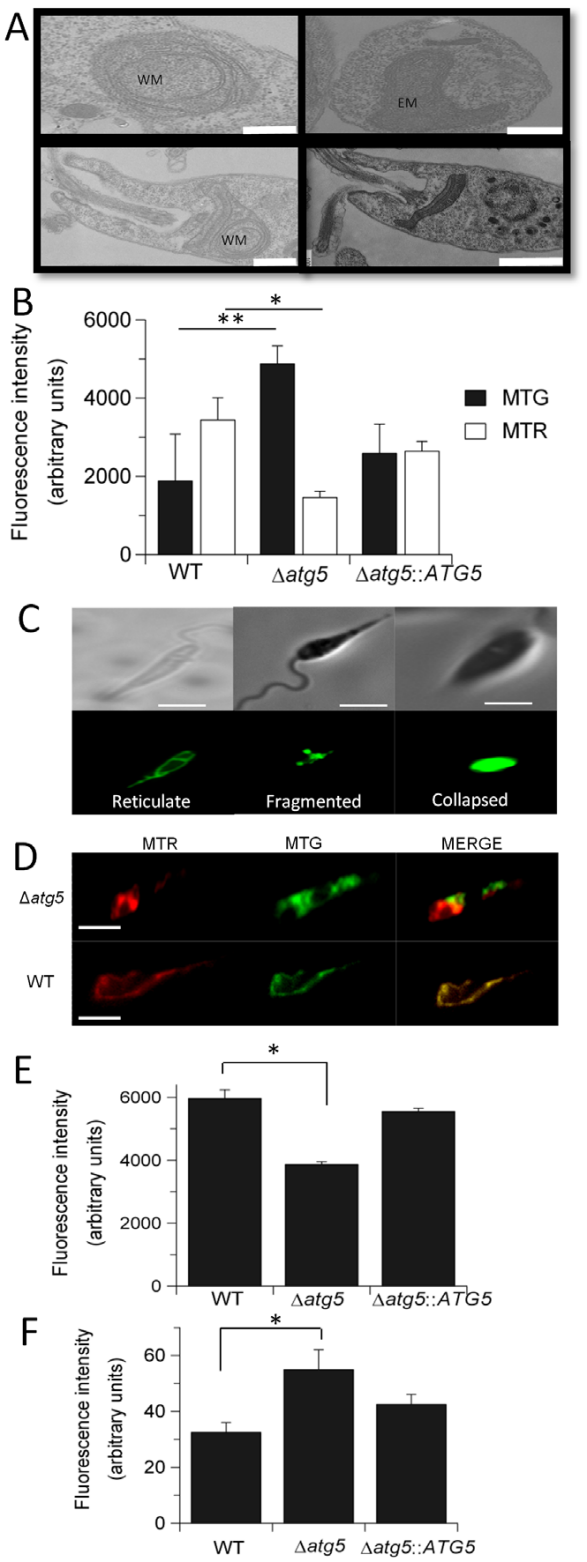
*L. major* Δ*atg5* promastigotes have a dysfunctional mitochondrion. (A) Enlarged (EM) and swollen (WM) mitochondria seen by transmission electron microscopy (TEM) in Δ*atg5* promastigotes under standard growth conditions. WT is shown at bottom right. Scale bar, 500 nm. (B) Fluorescent intensity from MitoTracker Red (MTR, 0.1 µM) and MitoTracker Green (MTG, 0.2 µM) in 2×10^6^ promastigotes after 30 min incubation at 26°C. Values shown are the means ± SD from three independent experiments. * and **, fluorescence was significantly different between WT and Δ*atg5* (p<0.05). (C) Types of mitochondrial morphology observed by fluorescence microscopy of Δ*atg5* promastigotes expressing the mitochondrial marker protein MUP-GFP. Scale bar, 10 µm. (D) Differential staining of promastigotes with both MTR (0.1 µM) and MTG (0.2 µM). Scale bar, 10 µm. (E) Viability, as measured by Alamar Blue reduction, of promastigotes. All data are means ± SD from three independent experiments. *, Alamar blue reduction was significantly different (p<0.05). (F) Spectrometric analyses of the DCF fluorescence intensity resulting from incubating promastigotes at 2×10^6^/ml with H2DCFDA at 0.1 mM for 2 h at 26°C. Values shown are the means ± SD from three independent experiments. *, DCF fluorescence was significantly different (p<0.05).

Transmission electron microscopy revealed the mitochondrion in Δ*atg5* to be swollen with an extended membranous structure (Figure 5A). This was suggestive of an increased mitochondrial mass, which was confirmed by MitoTracker Green (MTG) labelling (Figure 5B, solid bars; MTG is a green-fluorescent mitochondrial stain which localizes to mitochondria regardless of mitochondrial membrane potential). The additional membranes that were apparent also suggested an increased lipid content. Analysis of Δ*atg5* expressing MUP-GFP revealed a variety of mitochondrial morphologies (Figure 5C) that ranged from the reticular network characteristic of WT promastigotes (∼30% of the cells; left panel), through fragmented forms (∼25%; centre panel), to swollen mitochondrion with little apparent structure (∼45%; right panel). To investigate if mitochondrion function was compromised, the cells were stained with MitoTracker Red (MTR; this is a red-fluorescent dye that stains mitochondria in live cells and the accumulation of which is dependent upon membrane potential). Δ*atg5* were found to have less than half of the MTR fluorescence compared with the WT promastigotes (Figure 5B, open bars), indicating a loss of mitochondrial membrane potential. This was confirmed by co-staining the cells with MTR and MTG, which showed total co-localisation in the WT promastigotes but a lower degree of co-localisation in Δ*atg5* (Figure 5D). Alamar Blue reduction was also less in Δ*atg5* than WT (Figure 5E), indicating reduced mitochondrial respiration in the mutant. Assessing the levels of reactive oxygen species (ROS) using 2′,7′-dichlorodihydrofluorescein diacetate (H2DCFDA, intracellular cleavage and oxidation of this to yield the highly fluorescent 2′,7′-dichlorofluorescein [DCF] is a measure of ROS) showed these to be higher in Δ*atg5* than WT promastigotes (Figure 5F). Together these results suggest mitochondrial dysfunction on several levels.

### Phospholipid accumulation in Δ*atg5* promastigotes

**Figure 6:**
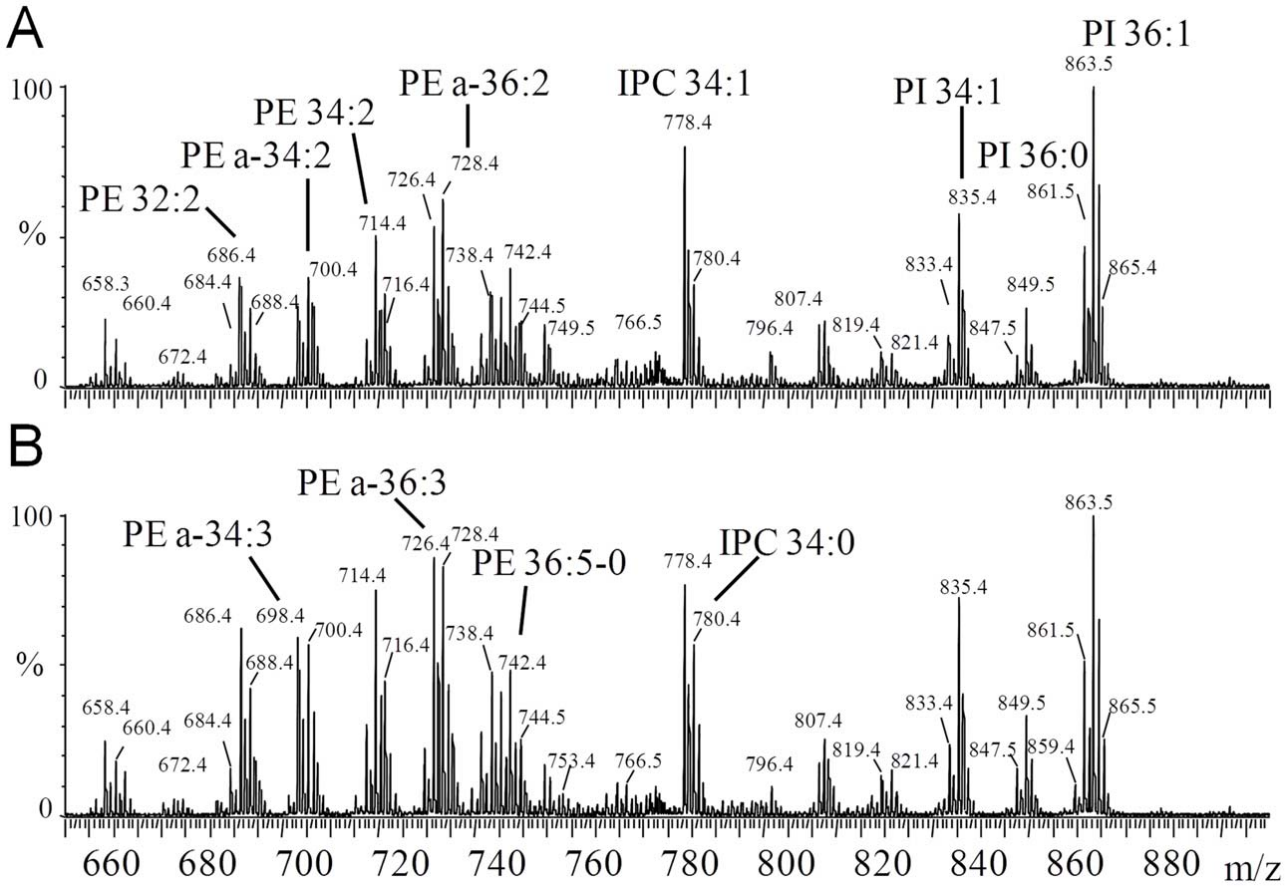
Phospholipid accumulation in Δ*atg5* promastigotes. Negative ion survey scans (650–900 m/z) of WT (A) and Δ*atg5* (B) promastigotes extracted for lipids and analysed by ES-MS, as described in Materials and Methods. a = (alkylacyl).

The mitochondrial changes resulting from deletion of *ATG5* were suggestive of effects upon lipid content and thus we compared the lipidome of Δ*atg5* and WT promastigotes cultured *in vitro* under standard growth conditions. The total intensity obtained from analysis of the extracted metabolites from 2×10^6^ promastigotes by liquid chromatography mass spectrometry (LC-MS) indicated that overall PE and phosphatidylcholine (PC) levels in Δ*atg5* were significantly higher (p<0.02) than the levels in the same number of WT promastigotes (PE, 3.4±1.3×10^7^ compared with 1.5±0.8×10^7^; PC, 1.1±0.4×10^8^ compared with 6.5±2.3×10^7^). Phosphatidylinositol (PI) and phosphatidylserine (PS) levels remained unchanged (data not shown). The apparent increase in PE and PC levels could be contributing to the increased membrane content of the mitochondrion in Δ*atg5*. As these data suggested a link between autophagy and phospholipid homeostasis of the cell, we investigated the phospholipid composition of WT and Δ*atg5* promastigotes in more detail using electrospray mass spectrometry. Survey scans using negative ion mode of the WT *L. major* between 600–900 m/z showed a wide range of molecular species from the three classes of phospholipid (Figure 6, all of the molecular species identified are detailed in Table S1). The major PE species between 680–745 m/z was the plasmalogen (alkenyl-acyl) at 726.4 and 728.4 m/z (for a-18∶1, 18∶2 and a-18∶1, 18∶1, respectively, where a = (alkylacyl) [38]) but the diacyl PE species was also identified at 714.4 m/z (for C34∶2). Several inositol phosphoceramide (IPC) species were observed between 680–810 m/z, the major species being the previously identified d16∶1, 18∶1-IPC at 778.4 m/z [39]. The third class of phospholipids detected were PIs, with an envelope of species between 800–900 m/z, the major species being at 835.4 and 863.5 m/z (diacyl 34∶1 and 36∶1, respectively). The equivalent negative ion survey scans for Δ*atg5* cells showed the presence of most molecular species identified in WT, but the majority of PE species increased significantly in Δ*atg5* cells relative to their WT counterpart (Figure 6, compare A and B). Large increases were apparent for PE species at 698.4, 726.4 and 738.4 m/z (a-34∶3, a-36∶3 and diacyl-36∶4, respectively) and IPC at 780.4 m/z for d16∶1, 18∶0 compared with the d16∶1, 18∶1 species at 778.4 m/z. In contrast to PE, no differences in any of the diacyl or alkenyl-acyl PI species or cardiolipin were obvious. More quantitative analysis of the overall PE levels in WT and Δ*atg5* promastigotes was facilitated by inclusion of an internal PE standard. Higher levels of the PE species (686–748 m/z) were clearly visible in the Δ*atg5* lipid extracts; normalisation using the internal standard indicated an approximate 2.5-fold increase in the total PE level compared with the WT promastigote levels (Figure S2). To investigate further how the observed increase in PE species in the Δ*atg5* cells could be due to the lack of ATG5 and autophagy, both WT and Δ*atg5* cells were grown in the presence of D_3_-serine prior to lipid extraction and analysis. As expected, D_3_-serine was incorporated into the phospholipid pool and manifested itself primarily in the IPC species [31]; the serine being utilised in *de novo* sphingolipid synthesis (Figure S3). Notably, the serine was not apparently incorporated into PE via decarboxylation of PS in either WT or Δ*atg5* promastigotes, as there was no detectable evidence of deuterated-PE with the same lipid moiety as the tiny amounts of detectable PS species (770–776 m/z, corresponding to a-36∶3 to a-36∶0) or of any other corresponding observable PE species, i.e. 686, 700, 714, 742 m/z. These data show that the only important route for PE synthesis in *L. major* is the Kennedy pathway.

### Δ*atg5* has a differentiation defect and reduced infectivity

**Figure 7:**
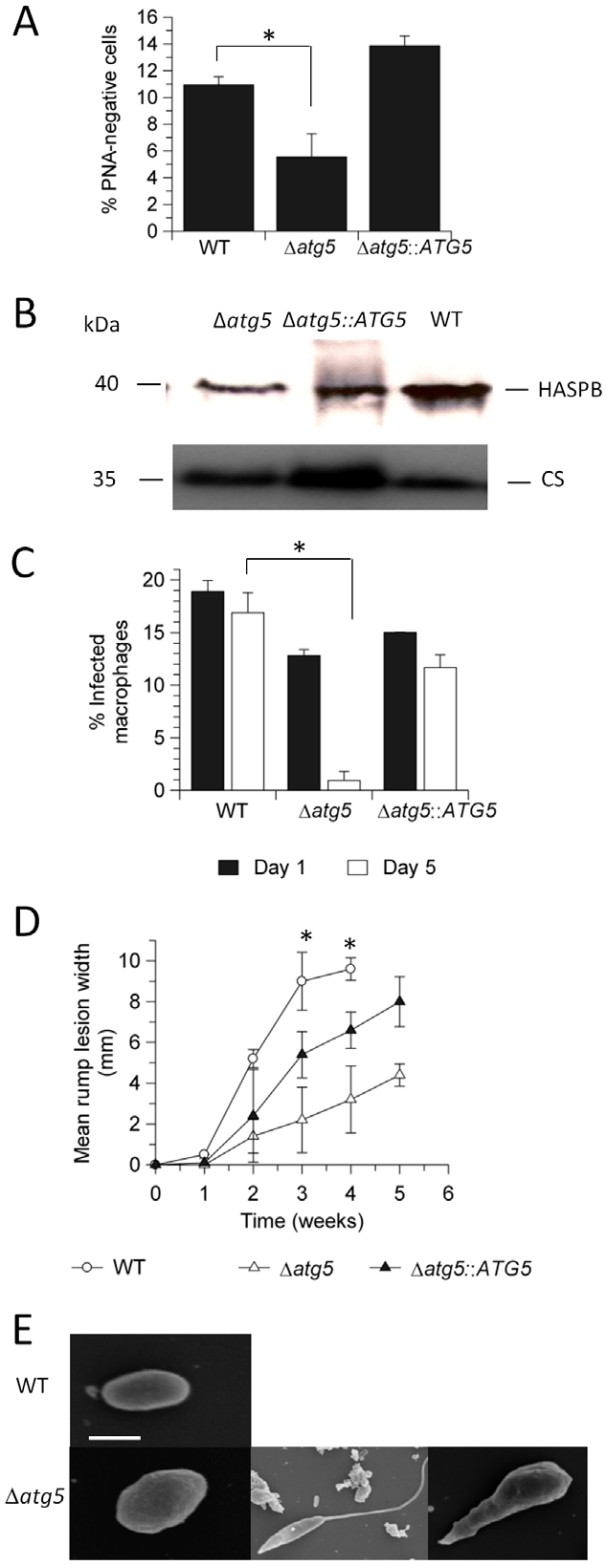
Promastigote differentiation and infectivity. (A) Proportion of metacyclic promastigotes in stationary phase cultures, assessed using the PNA assay. Values shown are the means ± SD from three independent experiments. *, differed significantly (p<0.05). (B) Western blot analysis of extracts of 10^7^ promastigotes at stationary phase of growth probed with α-HASPB. α-Cysteine Synthase was used as a loading control [56]. (C) Infectivity and survival of promastigotes in peritoneal macrophages *in vitro*, infected at a ratio 5∶1, with the infection rates being assessed after 1 and 5 days. *, differed significantly (p<0.01). (D) Lesion progression in BALB/c mice inoculated with 5×10^5^ stationary phase promastigotes. Values shown are the means ± SD from 5 mice. *, infection level of Δ*atg5* differed significantly from WT (p<0.01) and Δ*atg5*::*ATG5* (p<0.05). (E) Morphologies of cells isolated from mouse lesions and analysed by SEM. Scale bar: 2 µm.

We applied several approaches to investigate whether differentiation from procyclic promastigote to metacyclic promastigote and infectivity is impaired in Δ*atg5*. We found firstly that peanut agglutinin-negative metacyclic promastigotes [40] were less abundant in Δ*atg5* promastigotes than in the WT line (Figure 7A). Secondly, Δ*atg5* expressed lower levels of the metacyclic marker protein HASPB [41] than WT promastigotes (Figure 7B). Thirdly, Δ*atg5* promastigotes were taken up into macrophages to a similar extent as WT, but survived poorly intracellularly with most macrophages being cleared of parasites by day 5 (Figure 7C). This reduced virulence of Δ*atg5* was also evident *in vivo*, inoculation of Δ*atg5* promastigotes into mice generated rump lesions that were significantly smaller than those inoculated with WT promastigotes or the re-expressor line at weeks 3 and 4 (p<0.01 and p<0.05, respectively at each time point; Figure 7D). Analysis of parasite morphology by scanning electron microscopy showed that the parasites isolated from an infected mouse were predominantly amastigotes (∼88%) with sizes ranging from ∼2–4 µm with no apparent morphological differences from WT (Figure 7E, compare panels on left). Surprisingly, ∼12% of Δ*atg5* had a spindle-shaped body that was 6–10 µm in length and 75% of these had no external flagellum (Figure 7E, panels in centre and on right).

### Δ*atg5* occurs as abnormal promastigotes with reduced flagellar and body lengths

**Figure 8:**
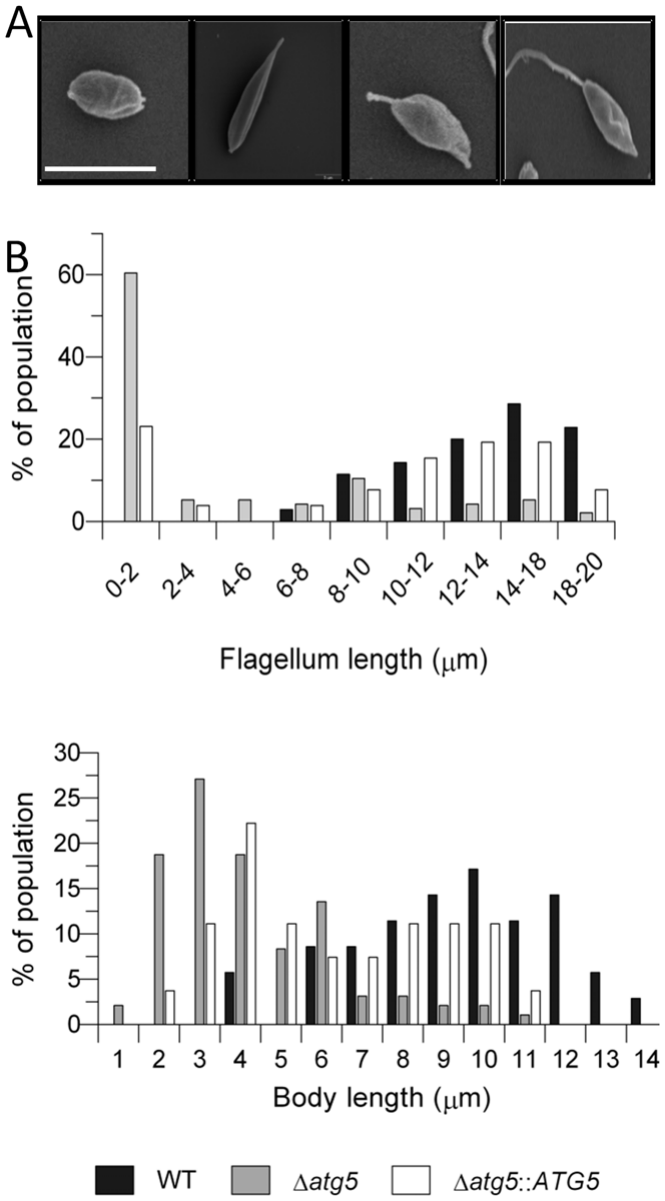
Morphology of Δ*atg5* promastigotes. (A) SEM analysis of promastigote culture initiated with Δ*atg5* isolated from a mouse lesion and cultured in nutrient-rich medium. Shown are ovoid and amastigote-like form (left); spindled-shaped form without an external flagellum (centre left panel) and with an external flagellum of varying lengths (centre right and right panels). Scale bar, 10 µm. (B) Distribution of flagella lengths and body lengths of stationary phase promastigotes. Data represent measurements from ∼200 cells from each promastigote population.

Amastigotes of Δ*atg5* were extracted from a mouse lesion and transformed *in vitro* to promastigotes which were used for all of the phenotypic characterization of Δ*atg5* in this study. The cells exhibited unusual morphological features and so we applied scanning electron microscopy to analyse the morphology of the cell population. Whilst many of the Δ*atg5* population on day 5 of *in vitro* culture were typical promastigotes, others were ovoid and amastigote-like and others were spindle-shaped, with or without an external flagellum (Figure 8A). Forms with no external flagellum represented ∼20% of the cells in logarithmic growth phase populations and their abundance increased to ∼60% in stationary phase of growth. Morphometric analysis of promastigotes of *L. major* in *in vitro* cultures reflected these differences and the mean flagellum and body lengths for stationary phase cells were significantly lower for Δ*atg5* parasites than for WT promastigotes (Figs. 8B); the mean flagellum lengths for WT, Δ*atg5* and Δ*atg5*::*ATG5* promastigotes were 13.7±3.1 µm, 4.7±2.1 µm, and 9.8±2.5 µm, respectively, and body lengths were 9.8±2.3 µm, 3.6±3.1 µm, and 7.6±5.9 µm, respectively. The mean body lengths and mean flagella lengths were significantly different between Δ*atg5* and WT (p<0.01 and p<0.05, respectively).

## Discussion

Homology-based genome annotation based on sequence similarity can lead to some interesting predictions on function, but the evolutionary distance between early and late eukaryotes means that predictions for protozoa need to be experimentally validated. A good example is the ATG12-ATG5 pathway in *Leishmania*. This was originally predicted by others to be absent [18] but subsequently possible putative homologues with very low sequence identity with yeast and human counterparts were identified by us and others [20], [42]. We have now resolved this uncertainty by demonstrating that the *L. major* ATG5-ATG12 conjugation system can be reconstituted *in vitro* using recombinant proteins. The conjugate was formed by the enzymatic reactions of ATG7 (E1-like) and ATG10 (E2-like) and required lys^185^ of ATG5, a free C-terminal glycine residue of ATG12 and ATP (Figure 2). The finding that ATG5 and ATG12 co-localise to puncta induced under starvation (and thus thought to be nascent autophagosomes) are consistent with these observations and support the hypothesis that the ATG5-ATG12 conjugation pathway exists within *L. major* promastigotes and apparently is, in the main, mechanistically similar to that of higher eukaryotes. Deletion of the *ATG5* gene from *Leishmania* generated mutants that were unable to form autophagosomes (as assessed by the absence of GFP-ATG8 puncta), which is entirely consistent with the ATG5-ATG12 conjugation pathway having a crucial role in autophagy in the parasite. More studies are required, however, to determine the extent to which the process in *Leishmania* is similar to that in yeast and mammalian cells and whether it shares features with the non-canonical processes of autophagy that are beginning to be elucidated [6]. In eukaryotes such as mammals, yeast and *Arabidopsis*, both the ATG5-ATG12 and the ATG8–PE conjugates localize at the phagophore to facilitate autophagosome genesis, but ATG5 and ATG12 are not normally observed on the completed autophagosome [5]. Our findings with *L. major* also show that the ATG5-ATG12 complex does not associate with all ATG8-labeled structures and is not apparently trafficked to the lysosome, consistent with the hypothesis that it assists in driving the expansion and/or curvature of the nascent autophagosome but dissociates from them just before, or immediately after, completion. One important unusual aspect of the process in *Leishmania*, however, is the apparent origin of the membrane and phospholipid for the phagophore, as we discuss below.

Our ability to generate mutants lacking *ATG5* confirmed that the protein is not essential for parasite survival *in vitro* as promastigotes or as amastigotes in macrophages or mice. Nevertheless, the survival of the Δ*atg5* parasites in explanted macrophages was very greatly reduced in comparison with WT parasites, as was growth in mice, so it appears that autophagy contributes in a very significant way, either directly or indirectly, to the parasite's virulence and so it should not be ruled out as a target for novel therapies. Autophagy is considered to be important for general cell homeostasis as well as for survival against adverse conditions such as oxidative stresses [43] and it seems very likely that this holds for *Leishmania* too. Cells in which the normal mechanisms responsible for homeostasis are adversely affected are very likely to be less able to withstand challenges such as those to which *Leishmania* is exposed when entering a macrophage. Moreover, interference with the normal processes of differentiation between forms, in which we have shown autophagy plays an important part [1], would also adversely affect survival when entering a new host or host cell.

One major mechanism mediating the Δ*atg5* mutant's reduced ability to withstand stresses of infection is the significant perturbation of mitochondrial function, including a lower membrane potential and so energy production and an enlarged mitochondrial mass, resulting from deletion of the gene encoding ATG5. Global analyses of the metabolome of the mutant revealed marked elevation of the phospholipid levels, in particular greatly elevated levels of PE and PC. Interestingly, levels of PI and cardiolipin (which occurs primarily in the inner mitochondrial membrane) were unaffected, showing specificity in the changes resulting from *ATG5* deletion. Phospholipid metabolism, especially that of PE which has many crucial signalling effects, is normally regulated very tightly within the mitochondrion and is inextricably linked to mitochondrial function, although relatively little is known about mechanisms regulating the phospholipid content and integrity of mitochondrial membranes [30]. However, all the evidence suggests that significant alterations to the mitochondrial PE composition would cause dysfunction of the organelle and result in a deficiency in ATP generation.

It seems very likely that the increased PE and PC content and the abnormal mitochondrial properties we observed for Δ*atg5* are causally linked. The key question, however, is how is ATG5 associated with these changes? As we have confirmed that ATG5 is necessary for autophagy in *Leishmania*, and as one known involvement of autophagy in mammalian cells and yeast is mitophagy, then one could postulate that the lack of the ATG5 in Δ*atg5* means that an important mechanism for removal of damaged and unwanted mitochondrial material is absent with resultant damage to the structure. The possibility that the lack of mitophagy is the cause of the observed mitochondrial dysfunction cannot be excluded, although there have been no definitive reports of mitophagy in *Leishmania* and we could not detect any mitochondrial proteins (using MUP-GFP as an outer membrane protein marker or ROM-GFP as an inner membrane protein marker) being trafficked to the lysosome in autophagosomes under our current experimental conditions. Moreover, the presence of a single large mitochondrion in *Leishmania* excludes mitophagy occurring as in yeast and mammalian cells in which whole mitochondria are enclosed within autophagosomes [22], [28]. Thus if mitophagy does occur in *Leishmania*, the mechanism must differ from that thought to operate normally in these other cells. In higher eukaryotes, mitophagy can occur co-ordinately with mitochondrial fission [44] and there must be a mechanism for mitochondrial fission in *Leishmania* in order to ensure correct partition of the single organelle during cell division; however, this type of mitophagy has not been observed to date. *Leishmania* must have mechanisms for maintaining mitochondrial performance and one similar to the recently discovered vesicular trafficking pathway between mitochondria and lysosomes in mammalian cells, that is independent of ATG8 and ATG5, is also worthy of consideration as being complementary to mitophagy [45].

There are, however, other possible causes of the elevated PE and PC levels in Δ*atg5*. It has been suggested that *Leishmania* is different from most eukaryotes in that the only route for PE synthesis is the Kennedy pathway with PS decarboxylase (PSD) being unimportant even though *Leishmania* do possess a *PSD* gene [31], [32]. In contrast, mammals use mainly the mitochondrion-located PSD route [46], with the PE produced actively exported to other organelles [30]. The first two steps of the Kennedy pathway in yeast and mammalian cells are exclusively located in the ER with the final step, involving ethanolamine-phosphotransferase (EPT), being located in either the ER or mitochondrion. Interestingly, in *T. brucei*, a trypanosomatid closely related to *Leishmania*, the EPT is mitochondrial (Gibellini, F & Smith T.K unpublished). Thus in *T. brucei* PE is synthesised in the mitochondrion. This could very well be the situation with *Leishmania* too, with all PE being generated in the mitochondrion. We therefore hypothesised that a second way in which the mitochondrion interplays with autophagy in *Leishmania* is in the provision of membrane for the developing phagophore and also PE to anchor the ATG8 in the phagophore. In the absence of autophagosome genesis in Δ*atg5* this phospholipid utilisation would not occur - with the result that phospholipid homeostasis in the mitochondrion would be disrupted and mitochondrial function thus impaired. Our data obtained in this study on the localisation of autophagosomes and phospholipid content of Δ*atg5* support this hypothesis. The application of dual-labelling of promastigotes with MUP-GFP or ROM-GFP as mitochondrial markers and mC-ATG5 showed that approximately two thirds of the ATG5 puncta were apparently associated with the mitochondrion and the multiple puncta occurring early in starvation with mC-ATG5 labelling alone had a distribution consistent with mitochondrial association too (Figure 3E). It has been recently shown for mammals that the outer membrane of mitochondria can be the source of autophagosome PE [12], [13], but only under unusual circumstances. Our findings on the elevated PE and also PC of Δ*atg5* are consistent with the hypothesis that in *Leishmania* the mitochondrion is a normal source of membrane and particularly PE and PC for autophagosome biogenesis and thus in this way *Leishmania* apparently differs greatly from mammalian cells and yeast.

Thus our results with *Leishmania* show that the functioning of ATG5, as well as being essential for autophagy itself, is also crucial for mitochondrial homeostasis indirectly as autophagy plays an important role in maintaining phospholipid and especially PE homeostasis. We suggest that the possibility that this is a mechanism contributing to the maintenance of mitochondrial membrane integrity in other eukaryotes warrants further investigation.

Interestingly, PE biosynthesis in *Leishmania* is elevated in promastigotes undergoing metacyclogenesis [31], [32] - which is when autophagy is most prevalent and required [1]. Moreover, the Kennedy pathway which is central to the provision of PE in *Leishmania* promastigotes is supplied from sphingolipid metabolism [31]. It is notable that the *Leishmania* mutants deficient in sphingolipid biosynthesis had a differentiation defect [31], which is consistent with these mutants being unable to synthesise PE and thus autophagy being prevented. Thus this study on sphingolipid synthesis provides further evidence of association between PE synthesis, autophagy and differentiation; it would be interesting to investigate whether these mutants present phenotypic alterations similar to those of Δ*atg5*.

The present study has provided also insights into consequences of the mitochondrial dysfunction that results from lack of ATG5. The inability of the Δ*atg5* mutants to salvage materials via autophagy presumably adds to the energy deficiency resulting from the mitochondrial dysfunction, and these two limitations together mean that under resource-limiting conditions the Δ*atg5* cells needed to resort to extreme measures. That the mutants showed morphological abnormalities including much reduced flagellum length in promastigotes suggests that flagellum regression is a mechanism whereby the parasite reduces energy utilisation, or indeed releases additional energy, in time of nutrient stress. Such changes have been reported previously [47], our findings suggest that these changes in Δ*atg5* could also be a secondary response to energy-generation crises.

The greatly reduced virulence of the Δ*atg5* mutants could be mediated in a number of ways. These include the lack of autophagy hindering the parasite's ability to transform to amastigotes. However, the changes in the mitochondrion resulting from deletion of *ATG5*, mediated by the lack of removal of PE and PC and/or the absence of a type of mitophagy that is needed for maintaining mitochondrial homeostasis, seems very likely also to be a key causal factor. The low virulence of the autophagy-deficient line provides evidence that components of the autophagy machinery in *Leishmania* warrant consideration for drug discovery programmes.

## Materials and Methods

### Ethics statement

All animal procedures were undertaken in adherence to experimental guidelines and procedures approved by The Home Office of the UK government. All work was covered by Home Office Project Licence PPL60/3929 entitled ‘Mechanism of control of parasite infection’. All animal protocols received ethical approval from the University of Strathclyde Ethics Committee.

### Parasites

*L. major* (MHOM/IL/80/Friedlin) promastigotes (designated WT for this study) were routinely grown and handled as described previously [48]. In this study, early log, mid log and early stationary phases of promastigote growth normally corresponded to approximately 5×10^5^, 5×10^6^, and 1×10^7^ parasites/ml, respectively. To study the effects of differing conditions, promastigotes at 10^7^ cells/ml were washed and suspended in phosphate-buffered saline (PBS) for starvation or HOMEM either serum-free or supplemented with 10 or 20% (v/v) FCS. The required antibiotics were added to the cultures of the transgenic lines are as follows: hygromycin B (Sigma) at 50 µg/ml; phleomycin (Cayla, France) at 10 µg/ml; puromycin (Calbiochem) at 10 µg/ml; neomycin (G418, Geneticin, Life Technologies) at 25 µg/ml.

### Metacyclogenesis and infectivity of promastigotes to macrophages *in vitro* and mice

The occurrence of metacyclic promastigotes was assessed using peanut agglutinin (PNA) [40] and western blot analysis of markers for metacyclic promastigotes. Infectivity to peritoneal macrophages from CD1 mice was with stationary phase promastigotes at a ratio of 5 promastigotes per macrophage and incubation for up to 5 days at 32°C in 5% CO_2_/95% air. Non-phagocytosed promastigotes were removed after 24 h and parasite abundance within the macrophages were determined after staining with Giemsa. Infectivity to mice was determined using groups of 5 mice inoculated subcutaneously in the rump with 5×10^5^ stationary phase promastigotes; the width of the resulting lesion in the rump was measured.

### Amastigote isolation and transformation

Excised rump lesions of mice in cold PBS containing 50 µg/ml gentamycin (Sigma) were homogenised in a glass tissue grinder, large debris was removed (150×g for 1 min at 4°C), amastigotes sedimented (1700×g for 15 min) and suspended in complete HOMEM medium with 50 µg/ml gentamycin and then incubated at 25°C to back-transform the amastigotes to promastigotes or immediately fixed for scanning electron microscopic (SEM) analysis.

### Parasite extracts for metabolome and phospholipid profiling

Parasite metabolites were extracted and analysed using LC-MS as detailed previously [49]–[51]. For phospholipid profiling, promastigotes at mid log growth phase, cultured in complete HOMEM medium at 26°C in the absence or presence of D_3_-serine (CDN) for the final 24 h, were extracted according to [52] and analyzed by electrospray mass spectrometry. Samples suspended in chloroform/methanol (1/2 v/v) were analyzed with a Micromass LCT mass spectrometer equipped with nanoelectrospray source. They were loaded into thin-wall nanoflow capillary tips (Waters) and analyzed by ES-MS in both positive and negative ion modes using a capillary voltage of 0.9 kV and cone voltages of 50 V. Where necessary MS/MS daughter ion scanning was performed on a Micromass Quattro Ultima triple quadrupole or a ABSCIEX 4000 Q-Trap mass spectrometer equipped with nanoelectrospray source using argon or nitrogen as a collision gas, respectively, with collision energies between 35–70 V depending upon lipid class. Each spectrum encompasses at least 50 repetitive scans.

### Plasmids for recombinant protein expression in *Escherichia coli*

The open reading frames (ORFs) of *ATG7* (LmjF07.0010), *ATG10* (LmjF31.3105), *ATG5* (LmjF30.0980), *ATG3* (LmjF33.0295) and *ATG12* (LmjF22.1300) were amplified by PCR using gene-specific primers (Table S2A). All PCR assays using Taq and Tgo DNA polymerases as part of the High Fidelity PCR system (Roche) were carried out for 30 cycles of denaturation (94°C, 15 s), annealing (65°C, 15 s) and extension (72°C, 2 min) and products cloned into pET28a^+^ and verified by nucleotide sequencing (Dundee Sequencing Services). Plasmids were transformed into BL21(DE3) for recombinant protein expression. A mutant of ATG5, ATG5^K128A^, was obtained by site-directed mutagenesis (Strategene) using primers shown in Table S2A, while the truncated proteins, ATG12g and ATG8g were generated by PCR as described above.

### Plasmids for gene deletion and expressing tagged fusion proteins in promastigotes

The plasmid used to generate the *ATG5* null mutant was the *pGL345-HYG* plasmid [53] modified with fragments of the 5′ and 3′ UTRs flanking the ORF of *ATG5*. The 5′ (1.0 kb) and 3′ (1.1 kb) flanks amplified from a *L. major* genomic DNA template by PCR with primers modified with *Hin*dIII/*Sal*I and *Sma*I/*BgI*II restriction sites, respectively, required for cloning (detailed in Table S2B) were sequentially inserted into the appropriately pre-digested *pGL345-HYG* to give *pGL345ATG5-HYG5′3′*. The *pGL345ATG5-BLE5′3*′ plasmid was generated from plasmid *pGL345ATG5-HYG5′3′* by replacing the *Spe*I/*Bam*HI ORF of the hygromycin resistance gene with a *Spe*I/*Bam*HI ORF of the bleomycin resistance gene. The *ATG5* ‘add back’ construct modified at the C-terminus with a poly-histidine epitope and containing the *Bgl*II and *Bam*HI sites was cloned into the pRIB-Pur vector [54] to produce *p*RIB*-Pur-ATG5-His*. *L. major ATG5* was cloned into the extrachromosomal pNUS*-mCherrynH* vector to give pN-*mC-ATG5* whereas *ATG12* and the gene encoding the mitochondrial ubiquitin-like protein (MUP; LmjF26.2070) were cloned into the pNUS-*GFPnH* vector to give pN-*GFP-ATG12* and pNUS-*GFPcH* vector to give pN-*MUP-GFP*. The gene encoding the mitochondrial rhomboid (LmjF02.0430) was cloned into the extrachromosomal pNUS-*GFPcN* to give pN-*ROM-GFP*. The pN-*GFP-ATG8* construct has been described previously [1].

### Generation of *L. major* transgenic cell lines

The cassettes used for transfection of promastigotes were linearized by *Hin*dIII/*Bgl*II digestion and the excised cassette used for electroporation using the Nucleofactor system (Lonza) with the program V-033. Parasite populations recovered after transfection were cloned by serial dilution. Clonal populations of parasites resistant to hygromycin were obtained and two of these heterozygotes (Δ*atg5^+/−^*) were used for the second round of transfections with the *pGL345ATG5-BLE5′3*′ construct and parasites were clones by serial dilution. One null mutant clone (Δ*atg5*) was selected for further analysis. Lines re-expressing *ATG5* were generated by integration of the pRIB-*Pur-ATG5-His* plasmid cassette, excised after digestion with *Pac*I and *Pme*I and used for electroporation of Δ*atg5* promastigotes, to generate Δ*atg5*::ATG5. Cell lines expressing tagged fluorescence proteins were generated by electroporation of promastigotes with 15 or 30 µg of plasmid and selection using the appropriate antibiotic(s) to give, for example, Δ*atg5*[GFP-ATG8], the nomenclature of the *ATG5* null mutant expressing GFP-ATG8 (Table S2B).

### Southern blot analysis of transgenic lines

Genomic DNA from the Δ*atg5* clones was extracted and Southern blots performed as described previously [48]. DNA (5 µg) was digested with *Xho*I, fractionated by agarose gel electrophoresis, nicked, denatured, neutralized and blotted onto Hybond™-N^+^ membrane (Amersham Pharmacia) by capillary transfer. The probe was prepared from a 1100 bp *Hin*dIII/*Sal*I 3′ flank fragment of *pGL345ATG5-HYG5′3′*.

### Monitoring puncta formation and mitochondrion structure

For live imaging, promastigotes in complete HOMEM medium were mounted on coverslips and the occurrence of puncta were observed using either a Nikon TE2000S or a Delta Vision core (Image Solutions) inverted microscope equipped with FITC and mCherry filter sets. To investigate autophagy induced by starvation, promastigotes were incubated in PBS (designated nutrient-deprived medium, ND) at 2×10^7^ cells/ml for up to 2 h and monitored for puncta similarly. Images were processed using IPlabs 3.7 image processing software (BD Biosciences Bioimaging). The presence and number of puncta within the cells was assessed in at least 100 cells from each of 3 independent experiments. Promastigotes at 1×10^7^ cells/ml were incubated with either 0.1 µM MitoTracker Red CMXRos (MTR, Invitrogen) or 0.2 µM MitoTracker Green TM (MTG, Invitrogen) for 30 min at 26°C or co-stained with both MTR and MTG similarly. Promastigotes were then washed in PBS and either mounted on glass slides for analysis by fluorescence microscopy or assessed for fluorescence (MTR at excitation 579 nm, barrier filter 599 nm; MTG at excitation 490 nm, barrier filter 516 nm) using a microtitre plate reader.

### Use of H2DCFDA and Alamar Blue

For assessing the levels of ROS, promastigotes at 1×10^7^/ml were harvested by centrifugation, washed once in serum-free HOMEM, and 2×10^6^ cells in 200 µl were incubated with 0.1 mM H2DCFDA (Molecular Probes) for 2 h at 26°C and the fluorescence measured using a microtitre plate reader (excitation 380–420 nm, barrier filter 520 nm). To evaluate metabolic activity and cell viability, Alamar Blue (resazurine salt, Sigma) was added to a final concentration of 0.0125% to 4×10^6^ promastigotes/ml at log phase of growth for one hour and its reduction measured using the fluorescence microtitre plate reader (excitation 550 nm, barrier filter 590 nm).

### Protein expression and purification

Expression of *L. major* ATG proteins, using the plasmids described above, in BL21(DE3) *E. coli* was carried out overnight at 15°C after induction with 1–2 mM isopropyl-β-D-thiogalactopyranoside (IPTG). Recombinant proteins were purified using an affinity chromatography column (Qiagen) and eluants obtained using 1 M imidazole were dialysed at 4°C overnight as follows: ATG7, ATG3, ATG10 and ATG8 - 50 mm Tris/HCl pH 7.5, 150 mm NaCl with 2 mm dithiothreitol; ATG5 - 20 mm Tris/HCl pH 8.0, 500 mm NaCl. The histidine tags of all ATG proteins except ATG5 were excised using thrombin (Novagen). The cleaved histidine tag and thrombin were subsequently removed by nickel chelate and benzamidine-Sepharose (Sigma) affinity chromatography.

### Reconstitution of the *L. major* ATG5-ATG12 conjugation system

Purified ATG7, ATG10, ATG12g and His-ATG5, each at 0.1 µg/ml, were mixed in reconstitution buffer (50 mm Tris-HCl, pH 8.0, 100 mm NaCl, 2 mm dithiothreitol, 1 mm MgCl_2_, and 1 mm ATP) and the reaction mixture was incubated for 1 h at 30°C. The conjugation reaction was stopped by boiling in sodium dodecyl sulphate polyacrylamide gel electrophoresis (SDS-PAGE) sample buffer. Samples were resolved by SDS-PAGE and subjected to western blot analysis using appropriate antibody.

### Western blot analyses

Western blots were performed as previously described [48] with primary antibodies: the α-His (Promega) and α-GFP (Abcam) antibodies were used at 1∶1000 and 1∶100 dilutions, respectively, and their corresponding secondary antibodies were α-rabbit IgG-horseradish peroxidase (HRP) (Promega) at 1∶20000 and α-rat-HRP (Promega) at 1∶2500. α-HASPB rabbit antibodies (kindly provided by Professor Deborah Smith, University of York) were diluted 1∶5000. α-CS (cysteine synthase) antibodies [55] were used a loading control at 1∶5000.

### Electron microscopy analyses

Parasites were fixed with 2.5% glutaraldehyde in 0.1 M phosphate buffer, pH 7.4 for 40 min and processed for transmission electron microscopy (TEM) as described previously [56]. Sections (80 nm) were examined with the Zeiss 912 TEM. For scanning electron microscopy (SEM), fixed samples were dried prior to coating with a very thin film of gold/palladium before examination. Promastigote body and flagella lengths were measured using the ESI Vision 3.2 Image analysis software (Olympus Soft Imaging Solutions). The cell morphologies noted within the parasite population were classified into groups according to the following criteria: amastigote-like forms that were ovoid and lacking an emergent flagellum; spindle shaped promastigotes with varying flagella and body lengths; and promastigotes that were similar to WT promastigotes. A minimum of 200 cells was examined for each sample.

### Data processing

Experimental data from macrophage infections, mice infectivity and Alamar Blue assays were pooled for comparison using unpaired t-tests. A p value of <0.05 was used as the level of significance.

### Accession numbers

Gene accession numbers (http://www.genedb.org) of proteins used in this study are:

ATG3, LmjF33.0295; ATG5, LmjF30.0980; ATG7, LmjF07.0010; ATG8, Lmj19.1640; ATG10, LmjF31.3105; ATG12, LmjF22.1300; ubiquitin-like peptidase MUP, LmjF26.2070; serine peptidase rhomboid, LmjF04.0850.

## Supporting Information

Figure S1 **Generation and validation of Δ** ***atg5*** **.** (A) Schematic representation of the *ATG5* locus and the plasmid constructs used for gene replacement. Arrows and boxes indicate the *ATG5* and antibiotic resistance genes and the 3′ and 5′ flanking DNA sequences, respectively. The restriction enzymes used for the different constructs and the expected sizes of fragments after *Xho*I digestion are shown. Key: *5*′*-DHFR* and *3*′-*DHFR*, dihydrofolate reductase flanking regions; *BLE*, bleomycin resistance gene; *HYG*, hygromycin resistance gene. (B) Southern blot analysis of genomic DNA digested with *Xho*I, separated on a 1% agarose gel, blotted onto a nylon membrane and hybridized with an alkaline phosphatase-labelled DNA probe corresponding to the 3′-flanking region of *ATG5*. The resistance genes are labelled. Molecular size markers are shown on the left (kb).
(TIF)

Figure S2 **Comparison of PE species in WT and Δ** ***atg5*** **promastigotes.** Negative ion ES-MS survey scans (600–1000 m/z) of lipid extracts with the addition of an internal standard PE (28∶0) from WT (A) and Δ*atg5* (B) promastigotes. Inserts are ESI-MS-MS positive ion spectra of neutral loss 141 m/z, the internal standard PE (28∶0) is indicated with by IS and an arrow.
(TIF)

Figure S3 **Analysis of D_3_-Ser incorporation into phospholipids in** ***L. major*** **promastigotes.** To investigate if the observed increase in PE species in the Δ*atg5* promastigotes was generated by PS decarboxylase activity, both WT (A) and Δ*atg5* (B) promastigotes were grown in the presence of D_3_-serine prior to lipid extraction and analysis by negative ion ES-MS survey scans (650–900 m/z).
(TIF)

Table S1 **Phospholipid species in** ***L. major*** **.** Lipids extracted from *L. major* were analyzed by ES-MS and subjected to MS/MS daughter ion spectra where necessary and assigned structures based upon their fragmentation ions and previous literature characterisations.
(DOC)

Table S2 **Plasmids and primers used in this study.**
(DOC)
