# Supplementary figures and images for: ATG5 Is Essential for ATG8-Dependent Autophagy and Mitochondrial Homeostasis in Leishmania major
Source: PLoS Pathog. 2012 May 17;8(5):e1002695. doi: 10.1371/journal.ppat.1002695 (PMC3355087; doi:10.1371/journal.ppat.1002695)

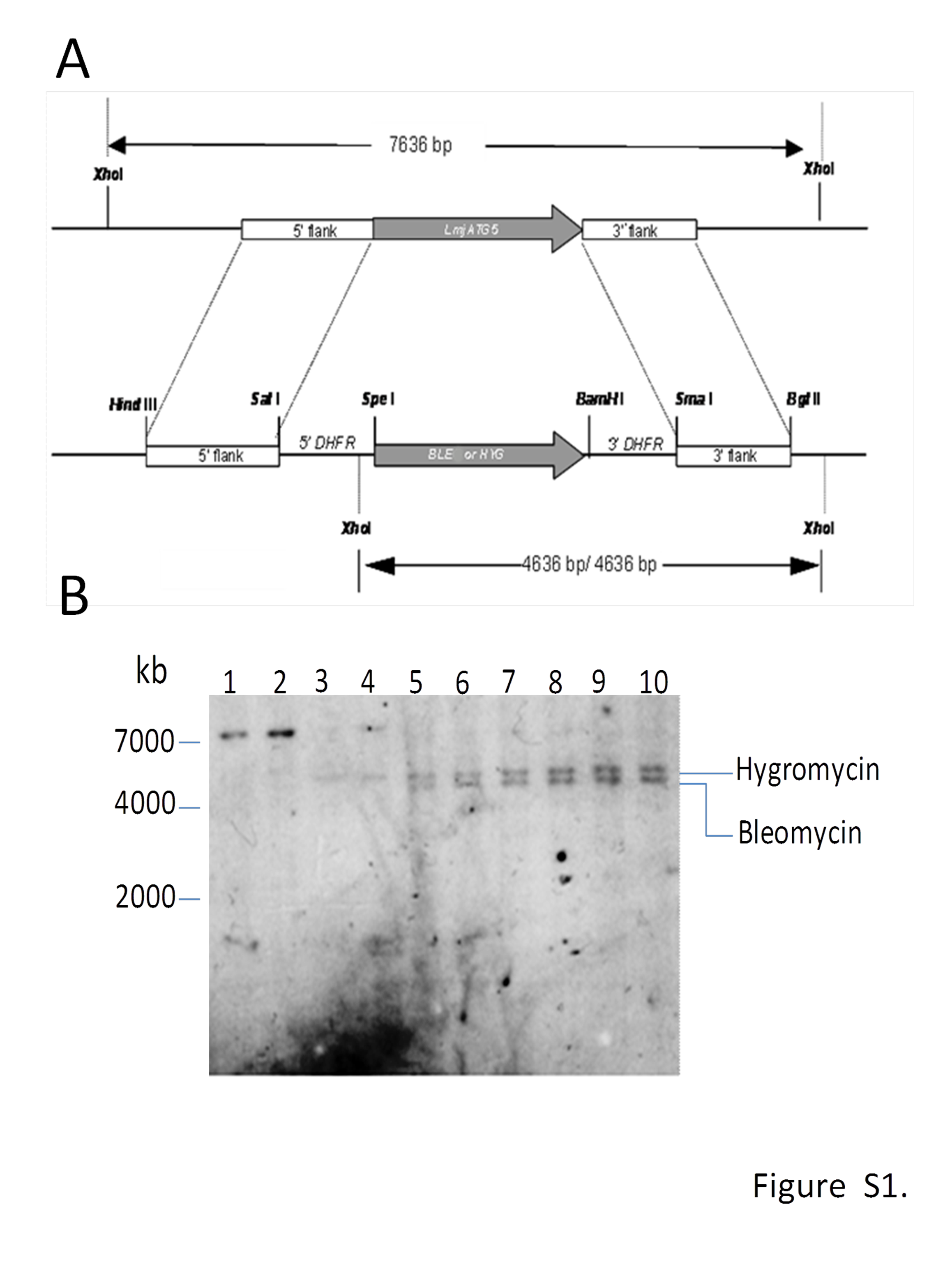

Supplement: Figure S1 — Generation and validation of Δ atg5 . (A) Schematic representation of the ATG5 locus and the plasmid constructs used for gene replacement. Arrows and boxes indicate the ATG5 and antibiotic resistance genes and the 3′ and 5′ flanking DNA sequences, respectively. The restriction enzymes used for the different constructs and the expected sizes of fragments after XhoI digestion are shown. Key: 5′-DHFR and 3′-DHFR, dihydrofolate reductase flanking regions; BLE, bleomycin resistance gene; HYG, hygromycin resistance gene. (B) Southern blot analysis of genomic DNA digested with XhoI, separated on a 1% agarose gel, blotted onto a nylon membrane and hybridized with an alkaline phosphatase-labelled DNA probe corresponding to the 3′-flanking region of ATG5. The resistance genes are labelled. Molecular size markers are shown on the left (kb). (TIF) [file ppat.1002695.s001.tif]

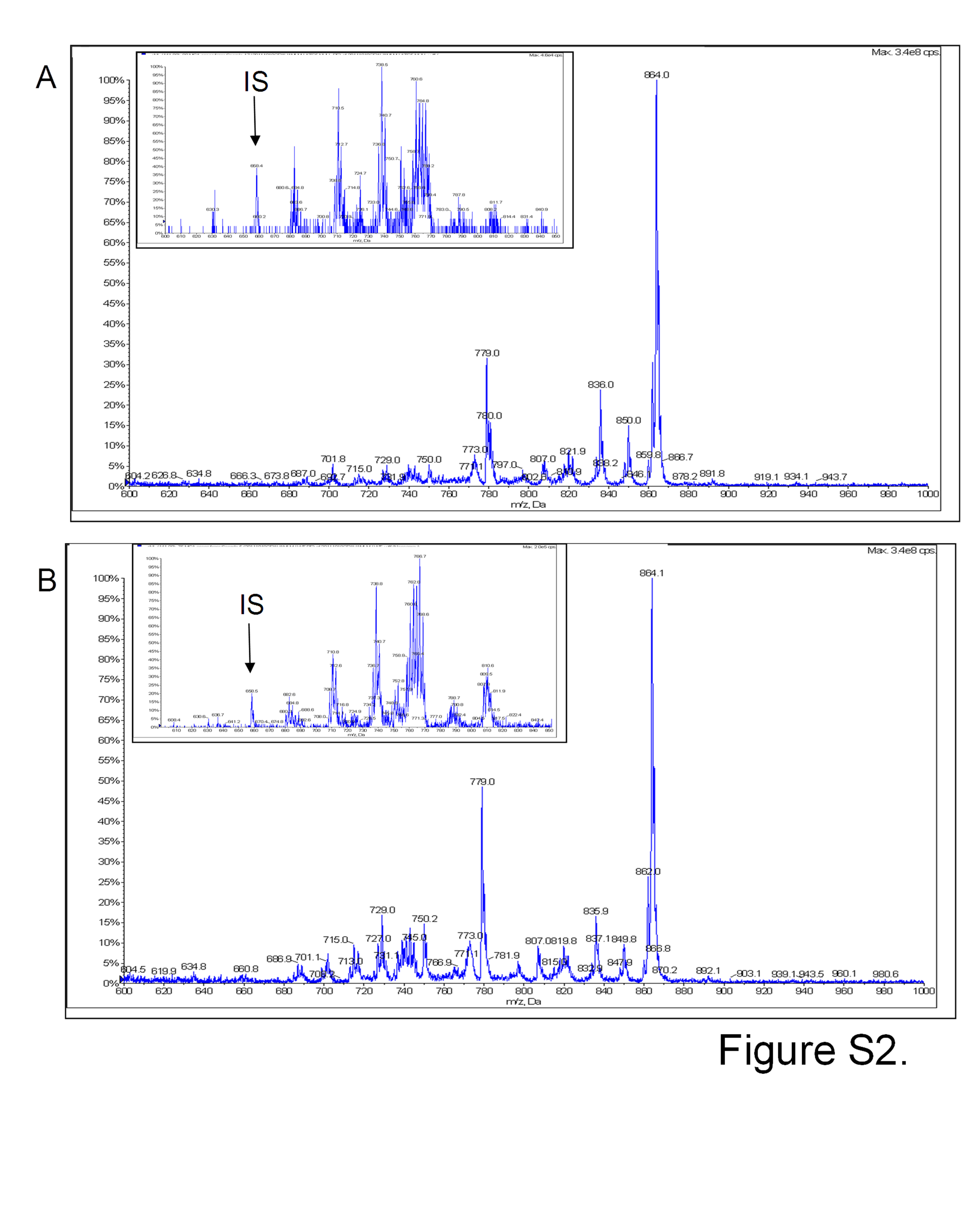

Supplement: Figure S2 — Comparison of PE species in WT and Δ atg5 promastigotes. Negative ion ES-MS survey scans (600–1000 m/z) of lipid extracts with the addition of an internal standard PE (28∶0) from WT (A) and Δatg5 (B) promastigotes. Inserts are ESI-MS-MS positive ion spectra of neutral loss 141 m/z, the internal standard PE (28∶0) is indicated with by IS and an arrow. (TIF) [file ppat.1002695.s002.tif]

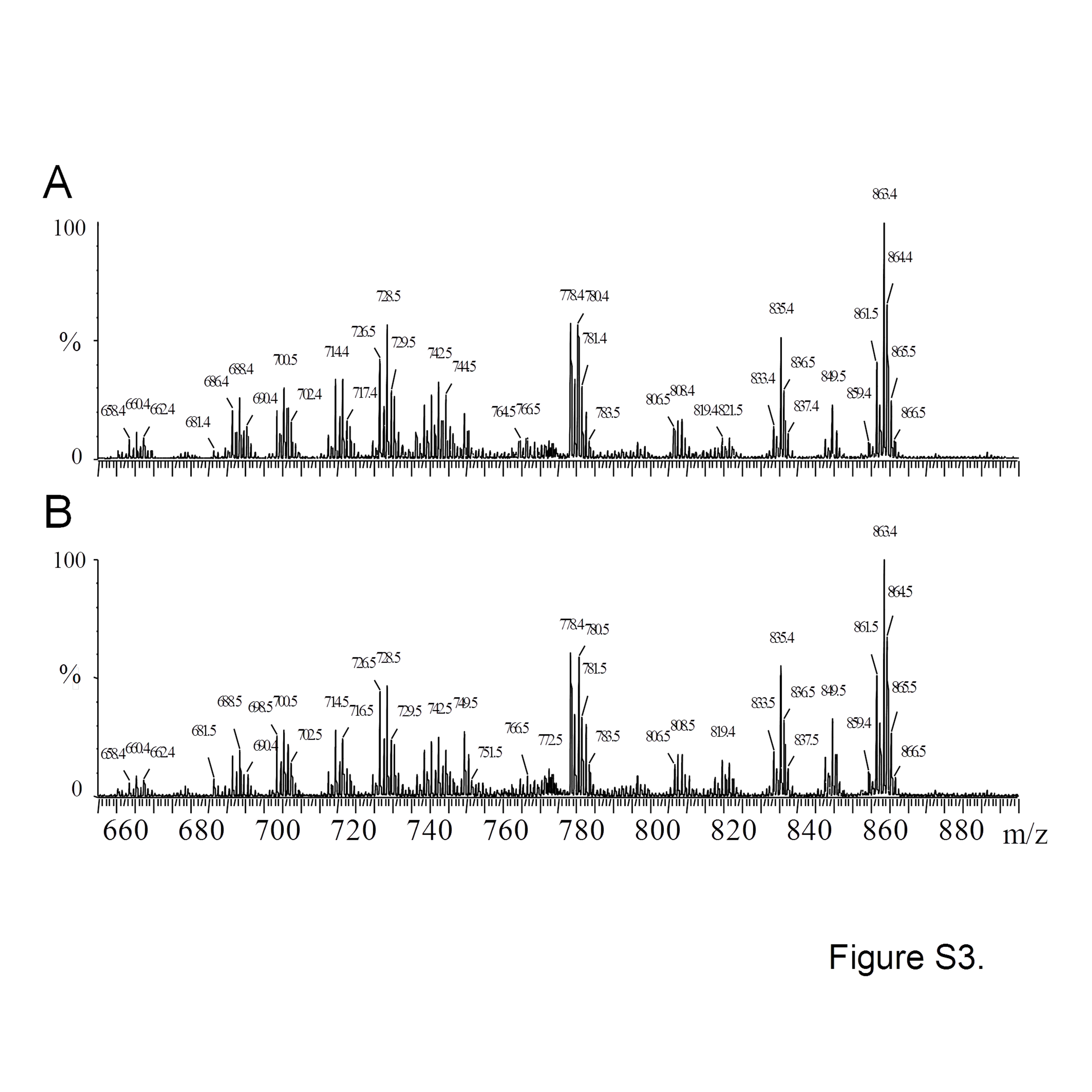

Supplement: Figure S3 — Analysis of D3-Ser incorporation into phospholipids in L. major promastigotes. To investigate if the observed increase in PE species in the Δatg5 promastigotes was generated by PS decarboxylase activity, both WT (A) and Δatg5 (B) promastigotes were grown in the presence of D3-serine prior to lipid extraction and analysis by negative ion ES-MS survey scans (650–900 m/z). (TIF) [file ppat.1002695.s003.tif]
