# Supplementary material for: ATG5 Is Essential for ATG8-Dependent Autophagy and Mitochondrial Homeostasis in Leishmania major
Source: PLoS Pathog. 2012 May 17;8(5):e1002695. doi: 10.1371/journal.ppat.1002695 (PMC3355087; doi:10.1371/journal.ppat.1002695)
Supplement: Table S1 — Phospholipid species in L. major . Lipids extracted from L. major were analyzed by ES-MS and subjected to MS/MS daughter ion spectra where necessary and assigned structures based upon their fragmentation ions and previous literature characterisations. (DOC) [file ppat.1002695.s004.doc]

**Table S1: Mass spectrometric analysis of phospholipid species in *L. major***

**PHOSPHATIDYLCHOLINE SPECIES**

| Peak^a^ | *m/z*^b^ | Lipid component^c^ | Principal component^d^ |
| --- | --- | --- | --- |
| 1 | 698.5 | 30:4 | 14:2/16:2 |
| 2 | 700.4 | 30:3 | 14:1/16:2 |
| 3 | 702.4 | 30:2 | 14:0/16:2 |
| 4 | 722.3 | 32:6 | 14:2/18:4 |
| **5** | **724.4** | **32:5** | **14:2/18:3** |
| 6 | 726.4 | 32:4 | 16:2/16:2 |
| 7 | 728.5 | 32:3 | 16:1/16:2 |
| 8 | 730.5 | 32:2 | 16:0/16:2 |
| 9 | 738.4 | e-34:4, a-34:5 | a-14:0/20:5 |
| 10 | 740.4 | e-34:3, a-34:4 | a-14:0/20:4 |
| 11 | 742.5 | e-34:2, a-34:3 | a-14:0/20:3 |
| 12 | 744.6 | e-34:1, a-34:2 | a-16:0/18:2 |
| **13** | **750.3** | **34:6** | **14:1/20:5** |
| **14** | **752.4** | **34:5** | **14:0/20:5** |
| 15 | 754.4 | 34:4 | 16:2/18:2 |
| 16 | 756.5 | 34:3 | 16:1/18:2 |
| 17 | 758.5 | 34:2 | 16:0/18:2 |
| 18 | 760.5 | 34:1 | 16:0/18:1 |
| 19 | 762.6 | 34:0 | 16:0/18:0 |
| 20 | 766.5 | e-36:4, a-36:5 | e-16:1/20:4 |
| 21 | 768.5 | e-36:3, a-36:4 | a-16:0/20:4 |
| 22 | 772.4 | e-36:1, a-36:2 | a-18:0/18:2 |
| 23 | 774.4 | e-36:0, a-36:1 | a-18:0/18:1 |
| 24 | 776.5 | a-36:0 | a-18:0/18:0 |
| **25** | **778.4** | **36:6** | **16:2/20:4** |
| **26** | **780.4** | **36:5** | **16:1/20:4** |
| **27** | **782.5** | **36:4** | **18:2/18:2** |
| 28 | 784.5 | 36:3 | 18:1/18:2 |
| 29 | 786.5 | 36:2 | 18:1/18:1 |
| 30 | 788.5 | 36:1 | 18:0/18:1 |
| 31 | 798.4 | e-38:2, a-38:3 | e-18:1/20:2 |
| **32** | **800.4** | **e-38:1, a-38:2** | **a-18:0/20:2** |
| **33** | **802.4** | **e-38:0, a-38:1** | **a-18:0/20:1** |
| **34** | **804.4** | **38:7** | **16:1/22:6** |
| **35** | **806.5** | **38:6** | **16:0/22:6** |
| 36 | 808.5 | 38:5 | 16:0/22:5 |
| 37 | 810.5 | 38:4 | 18:0/20:4 |
| 38 | 824.4 | e-40:3, a-40:4 | a-18:0/22:4 |
| 39 | 826.5 | e-40:2, a-40:3 | e-18:1/22:2 |
| **40** | **828.5** | **e-40:1, a-40:2** | **a-18:0/22:2** |
| **41** | **830.5** | **40:8** | **18:2/22:6** |
| 42 | 832.5 | 40:7 | 18:1/22:6 |
| 43 | 834.4 | 40:6 | 18:0/22:6 |
| 44 | 836.6 | 40:5 | 18:0/22:5 |
| 45 | 848.4 | e-42:6 | e-18:1/24:6 |
| **46** | **850.4** | **e-42:5** | **e-18:1/24:4** |
| **47** | **852.4** | **a-42:4** | **a-18:0/24:4** |
| **48** | **854.4** | **a-42:3** | **a-20:1/22:2** |
| 49 | 856.6 | a-42:2 | a-20:0/22:2 |
| 50 | 876.6 | 44:13 | 20:6/24:7 |
| 51 | 878.6 | 44:12 | 20:6/24:6 |
| 52 | 880.6 | 44:11 | 20:6/24:5 |

**PHOSPHATIDYLETHANOLAMINE SPECIES**

| Peak^a^ | *m/z*^b^ | Lipid component^c^ | Principal component^d^ |
| --- | --- | --- | --- |
| 1 | 634.4 | 28:0 | 14:0/14:0 |
| 2 | 656.4 | 30:3 | 14:1/16:0 |
| 3 | 658.3 | 30:2 | 14:0/16:2 |
| 4 | 660.4 | 30:1 | 14:0/16:1 |
| 5 | 662.4 | 30:0 | 14:0/16:0 |
| 6 | 684.4 | 32:3 | 16:1/16:2 |
| **7** | **686.4** | **32:2** | **16:1/16:1** |
| 8 | 688.4 | 32:1 | 16:0/16:1 |
| 9 | 690.4 | 32:0 | 16:0/16:0 |
| 10 | 698.4 | a-34:3 | e-16:1/18:2 |
| **11** | **700.4** | **a-34:2** | **a-16:0/18:2** |
| 12 | 702.4 | a-34:1 | a-16:0/18:1 |
| 13 | 710.4 | 34:4 | 16:1/18:3 |
| 14 | 712.4 | 34:3 | 16:1/18:2 |
| **15** | **714.4** | **34:2** | **16:0/18:2** |
| 16 | 716.4 | 34:1 | 16:0/18:1 |
| 17 | 718.4 | 34:0 | 16:0/18:0 |
| 18 | 724.4 | a-36:4 | a-18:1/18:3 |
| **19** | **726.4** | **a-36:3** | **a-18:1/18:2** |
| **20** | **728.4** | **a-36:2** | **a-18:1/18:1** |
| 21 | 730.5 | a-36:2 | a-18:1/18:1 |
| 22 | 736.4 | 36:5 | 14:0/22:5 |
| 23 | 738.4 | 36:4 | 18:2/18:2 |
| 24 | 740.4 | 36:3 | 18:1/18:2 |
| **25** | **742.4** | **36:2** | **18:0/18:2** |
| 26 | 744.5 | 36:1 | 18:0/18:1 |
| 27 | 746.5 | 36:0 | 18:0/18:0 |
| 28 | 748.5 | a-38:6 | e-18:1/20:5 |
| 29 | 750.5 | a-38:5 | e-18:1/20:4 |
| 30 | 762.5 | 38:6 | 18:2/20:4 |
| 31 | 764.5 | 38:5 | 18:1/20:4 |
| 32 | 766.4 | 38:4 | 18:0/20:4 |
| 33 | 768.4 | 38:3 | 18:1/20:2 |
| 34 | 770.4 | 38:2 | 18:0/20:2 |
| 35 | 772.4 | 38:1 | 18:0/20:1 |
| 36 | 796.4 | 40:3 | 18:1/22:2 |
| 37 | 820.4 | 42:5 | 18:0/24:5 |
| 38 | 822.4 | 42:4 | 18:0/24:4 |

**INOSITOLPHOSPHOCERAMIDE**

| Peak^a^ | *m/z*^b^ | Lipid component^c^ | Principal component^d^ |
| --- | --- | --- | --- |
| **1** | **778.4** | **IPC 34:1** | **d16:1/18:0** |
| **2** | **780.4** | **IPC 34:0** | **d16:1/18:1** |
| 3 | 806.5 | IPC 36:1 | d18:1/18:0 |
| 4 | 808.5 | IPC 36:0 | d18:1/18:1 |
| 5 | 832.4 | IPC 38:1 | d18:1/20:0 |
| 6 | 834.4 | IPC 38:0 | d18:1/20:1 |

**PHOSPHATIDYLINOSITOL**

| Peak^a^ | *m/z*^b^ | Lipid component^c^ | Principal component^d^ |
| --- | --- | --- | --- |
| 1 | 831.5 | 34:3 | 16:1/18:2 |
| 2 | 833.4 | 34:2 | 16:0/18:2 |
| **3** | **835.4** | **34:1** | **16:0/18:1** |
| 4 | 837.4 | 34:0 | 16:0/18:0 |
| 5 | 847.5 | e-36:1, a-36:2 | a-18:0/18:2 |
| 6 | 849.5 | e-36:0, a-36:1 | a-18:0/18:1 |
| 7 | 851.5 | a-36:0 | a-18:0/18:0 |
| 8 | 859.4 | 36:3 | 18:1/18:2 |
| **9** | **861.4** | **36:2** | **18:0/18:2** |
| **10** | **863.4** | **36:1** | **18:0/18:1** |
| 11 | 865.5 | 36:0 | 18:0/18:0 |
| 12 | 889.5 | 38:2 | 18:0/20:2 |
| 13 | 891.5 | 38:1 | 18:0/20:1 |
| 14 | 893.5 | 38:0 | 18:0/20:0 |

**PHOSPHATIDYLSERINE**

| Peak^a^ | *m/z*^b^ | Lipid component^c^ | Principal component^d^ |
| --- | --- | --- | --- |
|  |  |  |  |
| 1 | 769.4 | a-36:3 | a-18:1/18:2 |
| 2 | 771.4 | a-36:2 | a-18:0/18:2 |
| 3 | 773.5 | a-36:1 | a-18:0/18:1 |

1. All of the molecular species refers to the corresponding peaks in the positive and negative ion mode in Figures 5, S2 and S3. The peaks containing the most abundant species are in bold.
2. Observed [M+H]^+^ or [M-H]^-^ ions, mass over charge from survey scans as described in Materials and Methods.
3. Peak identities refer to total number of carbon atoms and double bonds. e = (alkenylacyl); a =(alkylacyl).
4. Only the principal component is given, in some instances as many as 5 individual species, with different combinations of acyl chains, result in the same total number of carbon atoms and double bonds. Precise fatty acyl constituents and their positions are given where possible, the most likely fatty acid candidates for the *sn*-1 and *sn*-2 constituents of the molecular species presented were deduced from the available literature.
