# Supplementary material for: ATG5 Is Essential for ATG8-Dependent Autophagy and Mitochondrial Homeostasis in Leishmania major
Source: PLoS Pathog. 2012 May 17;8(5):e1002695. doi: 10.1371/journal.ppat.1002695 (PMC3355087; doi:10.1371/journal.ppat.1002695)
Supplement: Table S2 — Plasmids and primers used in this study. (DOC) [file ppat.1002695.s005.doc]

**Table S2. Plasmids and primers used in this study**

Table S2A

| **For transforming *E. coli*** | | |
| --- | --- | --- |
| **Plasmids** | **Characteristics** | **Primers name and sequence (5’-3’)** |
| pET28a^+^-ATG3 | Plasmid containing a 1.16 kb ORF of LmjF33.0295 modified with the NdeI*/Hind*III restriction sites. | NT310:- 5’CATATGTCTCATCGGCGCTCTCTGTATGAA3’  NT311:- 5’AAGCTTTCACACTAAGTGAAACCCCGAGGA3’ |
| pET28a^+^-ATG5 | Plasmid containing a 1.38 kb ORF of LmjF30.0980 modified with the *Nde*1*/Hind*III restriction sites. | NT314:- 5’CATATGCTTCTCGCCATTGTGCGGGACCTCCTT3’  NT315:- 5’AAGCTTTCACACCTGAACCGTGACAAAAAT3’ |
| pET28a^+^-ATG5^(K128A)^ | Plasmid containing a lysine to alanine substitution at residue 128 of the 1.38 kb ORF of LmjF30.0980 | NT:-600:-5’CacatccggatggaggtgGCTcaggcacacaaggctgcc3’  NT:-601:- 5’ GGCAGCCTTGTGTGCCTGAGCCACCTCCATCCGGATGTG3’ |
| pET28a^+^-ATG7 | Plasmid containing a 2.18 kb ORF of LmjF07.0100 modified with the *Nde*I*/Hind*III restriction sites. | NT308:- 5’CATATGAGCGCCGTGAGCGGTAAACCGCAT3’  NT309:- 5’AAGCTTCTAGTCGGCCCACTCCTCGTCAGA3’ |
| pET28a^+^-ATG8g | Plasmid containing a 0.30 kb ORF of LmjF19.1630 modified with the *BamH*1*/Hind*III restriction sites. | NT 352:- 5’ GGATCCATGTCTTCCAGAGTAGCTGGGTCGTACAAG3’  NT 602:- 5’AAGCTTGaggacgggttcttgtacgtgacgtactctggcgagaacacgtacggc3’ |
| pET28a^+^-ATG10 | Plasmid containing a 0.77 kb ORF of LmjF31.3105 modified with the *Nde*I*/Hind*III restriction sites. | NT306:- 5’CATATGGACTGCGAGGCGGTCCTCGTG3’  NT307:- 5’AAGCTTTCATGCACGTGCGGTATCGAAATT3’ |
| pET28a^+^-ATG12 | Plasmid containing a 0.61 kb ORF of LmjF22.1300 modified with the *Nde*I*/BamH*I restriction sites. | NT442:- 5’GGATCCTCACCGCTGCACCGGTCGCCTCACCGTAT3’  NT444:- 5’ATGCATCATCATCATCATCATATGCACGCGCCACCACAGCCGCCGCCGCCACCT3’ |
| pET28a^+^-ATG12g | Plasmid containing a 0.6 kb ORF of LmjF22.1300 modified with the *Nde*I*/BamH*I restriction sites. | NT443:- 5’GGATCCTCAACCGCCGAAGGTGTTTTCCAGCAAGT3’  NT444:- 5’ATGCATCATCATCATCATCATATGCACGCGCCACCACAGCCGCCGCCGCCACCT3’ |

Table S2B

| **For tranfecting *L. major*** | | |
| --- | --- | --- |
| **Plasmids** | **Characteristics** | **Primers name and sequence (5’-3’)** |
| pNUS-*mC-ATG5* | Plasmid containing the 1.38 kb ORF of LmjF30.0980 modified with the *Bgl*II/*Kpn*I site for cloning into pNUS-*mCherrynH* vector | NT500:- 5’GGTACCTCAAGCGTAGTCTGGGACGTCGTATGGGT3’  NT501:- 5’AGATCTATGCGCTCGCAGAAGGAATACATCGGC3’ |
| pNUS-*GFP-ATG8* | Plasmid containing the 0.3 kb ORF of LmjF19.1630 modified with the *Bgl*II/*Xho*I site for cloning into pNUS-*GFPnH* vector |  |
| pN-*MUP-GFP* | Plasmids containing the 1 kb fragment from the ORF of LmjF26.2070 modified with *Nde*I/*Bgl*II for cloning into the pNUS-*GFPcH* vector | NT512:- 5’AGATCTGTTGCTCGCACCGCGCGCG3’  NT513:- 5’CATATGACGGCAGAGGCGGTGCCAGC |
| *pGL345ATG5HYG5’3’* | Plasmid derived from pGL345-HYG but modified with the 5’ flanks and 3’ flanks of ATG5 modified with *Hind*III/*Sal*I and *Sma*I/*Bgl*II restriction sites, respectively | NT447:- 5’AAGCTTCAACACCCTTCCCGGTTCCGC3’  NT448:- 5’GTCGACATGAGCAGGATGCAATACCACGACTG3’  NT445:- 5’CCCGGGTGATGTCAGCGAGGATTTCG3’  NT446:- 5’AGATCTAGCCGCCTCAGTGACCGACTGGCT3’ |
| pRIB-*Pur*-*ATG5-His* | Plasmid containing the ORF of ATG5 modified at the C-terminus with a poly-histidine tag and containing the *Bgl*II and *BamH*I restriction sites for cloning into the pRIB-Pur vector | NT:-516:- 5’GGATCCCATCATCATCATCATCATTCAAGCGTAGTCTGGGACGTCGTATGGGT3’  NT515-5’AGATCTATGCGCTCGCAGAAGGAATACATCGGC3’ |
| pNUS-*GFP-ATG12* | Plasmid containing the 0.63 kb ORF of LmjF22.1300 modified with the *Bgl*II/*Xho*I site for cloning into pNUS-*GFPnH* vector |  |
| pNUS-*ROM-GFP* | Plasmid containing the 1.2 kb ORF of LmjF04.0850 modified with the *Nde*I/*Kpn*I site for cloning into pNUS-*GFPcN* vector | OL2245:- 5’CCATATGCAGCAGCCATGCTTCTTTG3’  OL2246:- 5’TGGTACCGAGCGTGGCAGTGAGCTTGTCG3’ |

1. Besteiro S, Williams RA, Morrison LS, Coombs GH, Mottram JC (2006) Endosome sorting and autophagy are essential for differentiation and virulence of *Leishmania major*. J Biol Chem 281: 11384-11396.

2. Mottram JC, Souza AE, Hutchison JE, Carter R, Frame MJ, et al. (1996) Evidence from disruption of the *lmcpb* gene array of *Leishmania mexicana* that cysteine proteinases are virulence factors. Proc Natl Acad Sci U S A 93: 6008-6013.

3. Williams RA, Woods KL, Juliano L, Mottram JC, Coombs GH (2009) Characterization of unusual families of ATG8-like proteins and ATG12 in the protozoan parasite *Leishmania major*. Autophagy 5: 159-172.
